# Gene-Set Local Hierarchical Clustering (GSLHC)—A Gene Set-Based Approach for Characterizing Bioactive Compounds in Terms of Biological Functional Groups

**DOI:** 10.1371/journal.pone.0139889

**Published:** 2015-10-16

**Authors:** Feng-Hsiang Chung, Zhen-Hua Jin, Tzu-Ting Hsu, Chueh-Lin Hsu, Hsueh-Chuan Liu, Hoong-Chien Lee

**Affiliations:** 1 Institute of Systems Biology and Bioinformatics, National Central University, Zhongli, 32001, Taiwan; 2 Center for Dynamical Biomarkers and Translational Medicine, National Central University, Zhongli, 32001, Taiwan; 3 Department of Physics, Chung Yuan Christian University, Zhongli, 32023, Taiwan; 4 Physics Division, National Center for Theoretical Sciences, Hsinchu, 30043, Taiwan; Kyushu University, JAPAN

## Abstract

Gene-set-based analysis (GSA), which uses the relative importance of functional gene-sets, or molecular signatures, as units for analysis of genome-wide gene expression data, has exhibited major advantages with respect to greater accuracy, robustness, and biological relevance, over individual gene analysis (IGA), which uses log-ratios of individual genes for analysis. Yet IGA remains the dominant mode of analysis of gene expression data. The Connectivity Map (CMap), an extensive database on genomic profiles of effects of drugs and small molecules and widely used for studies related to repurposed drug discovery, has been mostly employed in IGA mode. Here, we constructed a GSA-based version of CMap, Gene-Set Connectivity Map (GSCMap), in which all the genomic profiles in CMap are converted, using gene-sets from the Molecular Signatures Database, to functional profiles. We showed that GSCMap essentially eliminated cell-type dependence, a weakness of CMap in IGA mode, and yielded significantly better performance on sample clustering and drug-target association. As a first application of GSCMap we constructed the platform Gene-Set Local Hierarchical Clustering (GSLHC) for discovering insights on coordinated actions of biological functions and facilitating classification of heterogeneous subtypes on drug-driven responses. GSLHC was shown to tightly clustered drugs of known similar properties. We used GSLHC to identify the therapeutic properties and putative targets of 18 compounds of previously unknown characteristics listed in CMap, eight of which suggest anti-cancer activities. The GSLHC website http://cloudr.ncu.edu.tw/gslhc/ contains 1,857 local hierarchical clusters accessible by querying 555 of the 1,309 drugs and small molecules listed in CMap. We expect GSCMap and GSLHC to be widely useful in providing new insights in the biological effect of bioactive compounds, in drug repurposing, and in function-based classification of complex diseases.

## Introduction

Microarray technique has been a powerful tool for profiling gene expression on a genome-wide scale and to study associations between gene expression and the pathology of common diseases, including various cancers and Alzheimer's disease [1, 2]. A common practice, the Individual Gene Analysis (IGA) of microarrays, focuses on statistics-based identification of differentially expressed genes (DEGs) between two phenotypes. Standard and popular methods of this type include student *t*-test, *z*-test, SAM, Limma, and ANOVA [3–7]. While most biological processes, including metabolic process, signal transduction, and regulation of transcription, typically involve the collaborative activation of large sets of genes, IGA methods emphasize the independence of individual genes and neglect the expected correlations in gene expression.

An improvement on IGA is to explore whether, among IGA-selected DEGs, functionally related gene sets, such as those given by Gene Ontology [8] and KEGG [9], are significantly expressed. An example of this approach is Fisher's exact test [10]. A drawback in this approach is that genes not among DEGs, namely the vast majority of genes, are excluded from the consideration. In the event when the DEG set is large, the correspondingly long list of sets of functionally related genes makes it cumbersome to compare results between studies. Most importantly, this approach tends to be dominated by large gene-sets, such as those of immune response and metabolic pathways, and results in the neglect of possibly important functions represented by smaller gene-sets.

The Connectivity map (CMap) was first developed as a generic solution for identifying the functional associations between diseases, genes, and drugs [11]. This approach provides a common analytical platform using genomic profiles as a shared language to connect diseases, gene functions, and drug activities. Many studies have employed disease-defined gene-sets to query CMap for the discovery of repurposed drug activities against common diseases, including diabetes [12] and Alzheimer's disease [13, 14], and solid tumours such as colon cancer [15], breast cancer [16], lung adenocarcinoma [17], and Inflammatory Bowel Disease [18]. CMap has also used to study drug-induced differential expression of drug target mRNA [19] and, in combination of public repositories of gene expression data characterizing diseases, to construct a database that connects input genomic profiles with CMap drugs and diseases [20]. The standard application of CMap has been IGA based [17]. However, results of IGA-based application of CMap on human samples tend to be dominated by cell types (Supporting Information in [11]). One way to overcome this tendency is to generate a consensus genomic profile for each drug by merging CMap data from different cell-lines [21]. In another approach, transcriptional response data (i.e., genomic profiles) of a drug is decomposed into factors specific to individual cell lines and factors shared by two or more cell lines, and only shared factors are assumed to be relevant in characterizing drugs [22].

Gene-Set Analysis (GSA) was developed to address the shortcomings of IGA [23]. GSA uses sets of genes connected by biological functions, instead of individual genes, as units of analysis. In Gene Set Enrichment Analysis (GSEA) [24], the first GSA method, the relative importance of a functional gene-set is represented by an enrichment score (ES). GSEA was employed to generate a map that links genomic profiles of diseases to corresponding drug responses in CMap [11].

More recent variants of GSEA, including GSA [25], SAFE [26], Catmap [27], ErmineJ [28], and SAM-GS [29], employ variations in matrix ranking, definition for enrichment scores, or scheme for significance estimation. Other methods including FunNet [30], PARADIGM [31], and COFECO [32] are network-based and more sophisticated, but their application may also be limited by the availability of gene-gene interactions. GSA methods have been employed to explore functional relationships in large-scale compendiums of clinical cancer cohort samples and to elucidate associations in drug-driven signatures for therapeutic purposes [18, 33]. Another unsupervised method based on annotation-driven clustering had also performed excellent results on recovering clinically relevant patient subgroups [34]. An integrated approach using chemical structures and biological functions to discover novel links between specific chemical structure properties and distinct biological responses in cells had also been reported [35].

In GSA, a genomic profile may be expressed as the set of ESs for a comprehensive list of gene-sets computed from that genomic profile; we shall call that set a functional genomic profile (hereafter, functional profile). Because a functional profile neither relies on an arbitrary threshold for gene selection, as does IGA, nor by definition is it dominated by a few functionalities involving large gene-sets, it is expected to be more accurate and sensitive in reflecting the global as well as detailed properties of a genome-wide gene expression than IGA.

**Fig 1:**
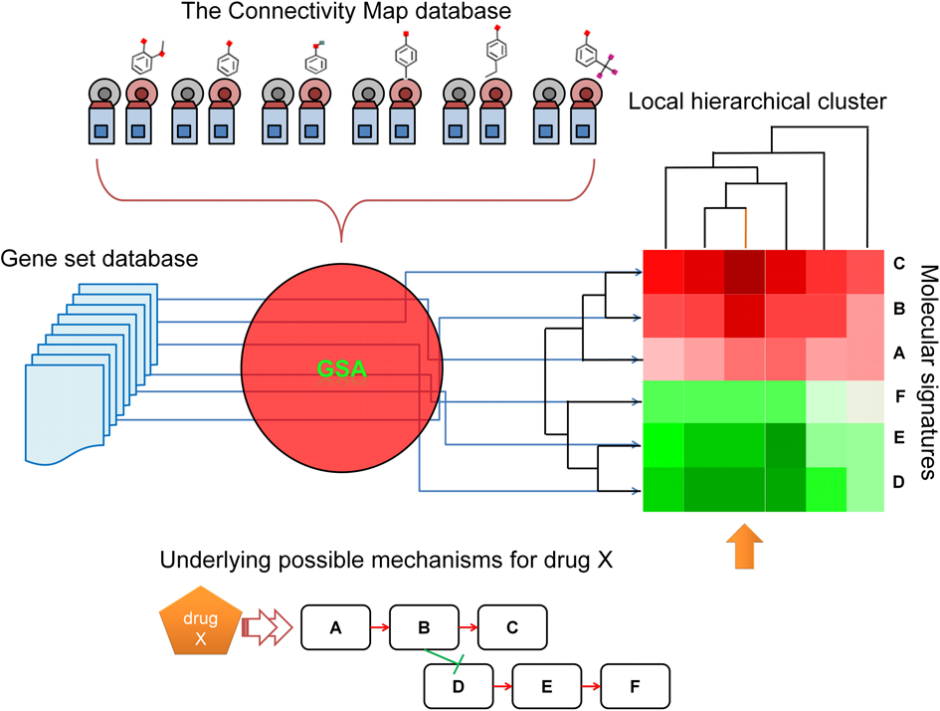
The GSLHC workflow.

Here, we built Gene-Set Connectivity Map (GSCMap), an enhanced version of CMap where the genomic profiles of drugs in the CMap database are converted to functional profiles. Like CMap, GSCMap may be used for repurposed drug discovery, except that in GSCMap the functional signature of a phenotype is matched to functional profiles of drugs. The goal is to construct a database that one may expect to yield a more robust drug-phenotype association. We conducted tests to establish the internal consistency of GSCMap. We showed that grouping of drugs with similar biological activities is much more robust with GSCMap than with CMap in IGA mode. For an application of GSCMap we developed Gene-set-based Local Hierarchical Clustering (GSLHC), which utilizes an agglomerative hierarchical method for clustering a subset of functional gene-sets associated with "local" drugs responses (Fig 1). The idea is that, given a very large matrix of gene-set enrichment scores, a clear pattern of coordinated expression in sets of functionalities are usually confined to a subgroup of samples, a pattern that may not be easily detected by global measurements [36, 37]. Through GSLHC we identified the therapeutic properties and putative targets of 18 compounds of previously unknown characteristics listed in CMap, placing each in a subclass of drugs grouped by the similarity of the functional response they induce. Eight of the 18 subclasses contain putative anti-cancer activities. Our results revealed novel links in terms of gene-sets, and drug-versus-functions.

Our results showed GSCMap to be a robust and biologically more reliable version of CMap, and GSLHC, in combination with GSCMap, to be useful in discovering linkages among bioactive compounds characterized by their functional properties.

## Materials and Methods

### External database

The CMap database (build 02). Four types of human cancer cell lines (MCF7, PC3, HL60, SKMEL5) were treated with 1,309 distinct small-molecules including U.S. Food and Drug Administration (FDA) approved drugs and uncharacterized bioactive compounds (call perturbagens by the authors of CMap, here simplicity referred as drugs), for a total of 6,097 treatments [11]. Gene (total RNA) expressions from the 6,097 “instances” (an instance is a cell line treated with a drug at a dosage, and its non-treated control) were recorded in two batches of microarrays: 671 HG-U133A (Affymetrix) chips (on 407 drugs) and 5,426 HT-HG-U133A chips (for a total of 6,097 chips on 1,309 drugs). Raw data were downloaded from the CMap website (http://www.broadinstitute.org/cmap/).

Molecular signature database. We downloaded the annotated 4,884 gene-sets (called tags) from the Molecular Signatures Database (MSigDB: http://www.broadinstitute.org/gsea/msigdb/index.jsp) [38]. We used four types of tags in MSigDB: C2: curated tags from known pathways, online databases, and knowledge of domain experts; C3: motif tags based on conservative cis-regulatory motifs from human, mouse, rat, and dog genomes; C4: computational tags determined by co-expression neighbourhoods centered on 380 cancer-related genes; C5: gene-ontology tags collected from the same GO annotations of genes. C1 (positional tags on each human chromosome) was not included in this study for saving the time on big size of tags. For convenience, gene symbols in each tag were combined and transformed in HG-U133A Affymetrix ID according to the updated annotation file from Affymetrix website (http://www.affymetrix.com/estore/).

Chemical structure database. In order to cluster compounds based on 3D structure similarity, we queried 1,309 drug names on NCBI PubChem database (http://pubchem.ncbi.nlm.nih.gov/). Next, the retrieved 1,267 compounds (97% of CMap databsets) were hierarchically clustered by *Chemical Structure Clustering* tool based on the 3D structure (fingerprint) similarity using the single linkage algorithm on PubChem website [39]. Finally, we partitioned the tree into K clusters with K ranging from 10 to 200, and evaluated the clustering performance using F-score [40].

Pharmacological classification system. We retrieved class information of 798 compounds (61% of CMap databsets) from the Anatomical Therapeutic Chemical (ATC) classification system in the World Health Organization (WHO) website (http://www.whocc.no/) for information on similar therapeutic classes. In this system, drugs are classified into groups at 5 different levels: the first level of code indicates the anatomical main group; the second level of code indicates the therapeutic main group; the third level of code indicates the therapeutic/pharmacological subgroup; the fourth level of code indicates the chemical/therapeutic/pharmacological subgroup; the fifth level of code indicates the chemical substance. We used the first four levels of ATC to evaluate the gene and tag clusters performance using F-score. The fifth level of the code was not included in our analysis because at this level CMap was too fragmented–almost one drug to a class–for the code to be useful.

Molecular target database. We extracted information on known therapeutic protein targets, relevant diseases or cancers, and corresponding drugs (787 drugs; 60% of CMap datasets) from the Therapeutic Target Database (TTD: http://bidd.nus.edu.sg/group/ttd/) [41]. The working types on specific targets by the corresponding drugs (including activator, adduct, agonist, antagonist, antibody, binder, blocker, breaker, cofactor, inducer, inhibitor, intercalator, modulator, multitarget, opener, regulator, stimulator, and suppressor) were simply divided into two major groups: inhibition or activation. Because drugs and targets do not have one-to-one correspondence, we did not calculate F-score based on the small class size. Instead, we computed drug-drug correlations by target group in IGA and GSA. The drug-pair is assumed to have correlation value of 1 if they have similar effects on the same protein target.

### Local database

CMap mirror database. Following the original methods described in CMap, the raw image of CEL files for the 6,097 instances from the CMap database were converted to average log-ratios and confidence calls using the algorithms MAS 5.0 (Affymetrix) and linear-fit-on-Pcall [11]. For each instance the log-ratios for the 22,283 HG-U133A probesets were ranked and the ranked data for all instances were saved in matrix form locally.

Local CMap program. The web version of CMap cannot be queried in batch mode. Furthermore, in each individual query the number of genes, or the size of the tag, is limited to 1000. To overcome these limitations, we used C++ language to build a local program encoding the same algorithms and datasets used by CMap. This program allows CMap-type queries to be made locally in single or batch mode, and permits GSEA (Gene Set Enrichment Analysis [38]) parameters be varied. The program was tested for reliability and speed before applied to the current study (see Results).

### Matrix CMap and the enrichment-score matrix GSCMap and their sub-matrices

**Cmap** is a 22,283x6,097 probe-set versus instance matrix; elements of matrix are log-ratios of expression intensities. From this a number of extend maps/matrices were constructed:

**Cmap1** 2013 The 22,283x671 sub-matrix of CMap involving the 671 instances in CMap v1.0.

**tCMap1** – A 300x671 sub-matrix of CMap1 involving the 300 highest variance probe-sets.

**CMd** – A 22,283x1,309 probe-set versus drug matrix reduced from CMap by averaging over same-drug instances.

**IGCMd** – A 4,884x1,309 sub-matrix of CMd involving the 4,884 highest variance probe-sets.

**GSCMap** – A 4,884x6,097 tag versus instance matrix; elements of the matrix are enrichment scores (ESs). For each of the 4,884 tags from MSigDB (collections C2-C5), we queried the 6,097 instances in CMap (version 2.0) to yield a 6,097-component vector (called Vd) of Kolmogorov-Smirnov statistic [11, 42, 43] based ESs, as defined in [38]. GSCMap is the set of 4,884 Vd’s.

**GSCMap1** – The 4,884x671 sub-matrix of GSCMap involving the 671 instances in CMap v1.0.

**tGSCMap1** – A 300x671 sub-matrix of GSCMap1 involving the 300 largest ES variances tags.

**GSCMd** – A 4,884x1,309 tag versus drug matrix. In CMap each drug were treated a variably multiple (averaging 6,097/1,309 = 4.66) times. For each tag and each drug the matrix element is the Kolmogorov-Smirnov statistic score (as in GSEA [38]) obtained by ranking the vector Vd corresponding to the tag and querying it using the multiple treatments for that drug.

### Significance by permutation and normalized enrichment score (NES)

We tested the significance of the ES of a tag-drug pair by random permutation. Given a tag and a drug, and suppose the drug had *t* treatments in CMap and an (tag versus drug) enrichment score *ES*_*0*_. We generated a distribution of randomized ESs by running *r* trials, in each trial recalculating the Kolmogorov-Smirnov ES by replacing the *t* treatments for the drug by *t* randomly selected treatments among the 6,097 treatments. A randomization (two-sided) *p*-value for the ES was computed from *ES*_*0*_ and the distribution. The normalized enrichment score (NES) was taken to be *ES*_*0*_ divided by the mean of the distribution [38]. In this work we set *r* = 10,000.

### Gene-set based Local Hierarchical Clustering (GSLHC)

GSLHC is an application of GSCMap for discovering links among drugs through tags strongly acted on by the drugs. Its implementation involves the steps: (i) Select a query drug set, which may be a single drug or a group of drugs with known shared property, or a drug of unknown property. (ii) For the query drug set, cull from GSCMap the functional profiles of drugs a subset of tags, each of which significantly enriched against every drug in the query drug set, where significant enrichment is determined by a threshold randomization *p*-value below an upper bound (we used *p* < 0.005). In the randomization test we generate a distribution of ESs by computing the ES for a tag-drug pair many times, each time replacing the genes in the tag by randomly selected genes from the entire gene pool [23]. (iii) Do a two-way hierarchical clustering of the culled tags with the entire set of 1,309 drugs, and cut out from the resulting heatmap the clade of drugs that includes the query drug set with correlation above a threshold value (we used 0.9).

### Cluster evaluation

We used the F-score, a harmonic mean of precision and recall [40], to evaluate a cluster as a classifier of a known classification. Let TP, FP, and FN be true positive, false positive, and false negative, respectively. The precision rate P and recall R rate of the cluster are respectively given by P = TP/(FP + TP) and R = TP/(TP+ FN). Suppose several nodes in a cluster are meant to represent a classification, then, for class *i*, the F-score F_i_ for that class is the maximum nodal value for 2PR/(P+R), and the F-score for the classification is the weighted average of F_i_ summed over the nodes. The higher the F-score, the better the classification by cluster. The F-score ranges from 0 to 1.

Whenever possible, computations were conducted in the R environment (R version 2.15.1). Conversion of CMap to GSCMap was lengthy and took many hours of computation time. However, a typical application of GSLHC for constructing a high-correlation drug cluster requires less than one minute on a standard student grade laptop.

### Ethic information

None.

## Results

### The local program reproduced results from CMap server with better efficiency

We used a tag called BRUINS_UVC_RESPONSE_LATE, which contains 1,137 genes differentially expressed only 12 h after UV-C irradiation of MEF cells, from MSigDB to compare the local program with the remote CMap server on the 100 drugs with the smallest *p*-value. The two programs yielded practically identical ESs (S1A Fig, dashed lines), and almost identical permutation *p*-values (S1A Fig, solid lines). Identical *p*-value were not expected; proportionally large differences in *p*-value occurred only when *p* < 10^−3^. We used the 772 tags in the C2 collection of MSigDB (number of genes in tags ranged from 50 to 1000) to compare the speed of the local program and the CMap server and found that the computation times were comparable, but the local program was slower when the gene number in the tag exceeded 600 (S1B Fig). The slower speed of the local program was more than compensated by the possibility of querying in batch mode.

### DEGs have low reproducibility in CMap genomic profiles

In CMap each of the 1,309 perturbagens has an average of 4.7 genomic profiles (from different treatments) resulting from the total of 6,097 treatments. We computed the fractional overlaps of top-1,000 DEGs between pairs of genomic profiles. The average reproducibility (common DEGs/1000) between different-perturbagen pairs has a sharp peak at 0.05, with few cases exceeding 0.1. That of the same-perturbagen pairs also peaks strongly at 0.06, but has a long weak tail (S2 Fig), with 10,771 of cases having a reproducibility greater than 0.2.

### The CMap and GSCMap matrices and their sub-matrices were constructed

Here, by CMap we mean the 22,283 (probes) x 6,097 (instances) matrix of log-ratios from CMap database. Using CMap we constructed the 4,884 (tags) x 6,097 GSCMap matrix of ESs using the 4,884 tags in MSigDB. Then we constructed sub-matrices of CMap, CMap1 (22,283x671), tCMap1 (300x671), CMd (22,283x1,309), and IGCMd (4,884x1,309), and sub-matrices for GSCMap, GSCMap1 (4,884x671), tGSCMap1 (300x671) and GSCMd (4,884x1,309), where 1,309 refers to the number of drugs/small molecules in CMap, 671 refers to the number of instances in CMap v1.0, 4,884 refers to the number of tags in MSigDB or the 4,884 highest variance probe-sets (for IGCMd), and 300 refers to 300 highest variance probe-sets (CMap) or ESs (GSCMap) (detail in Methods).

### Cell-type dependence of CMap data was strong in IGA but weak in GSA

**Fig 2:**
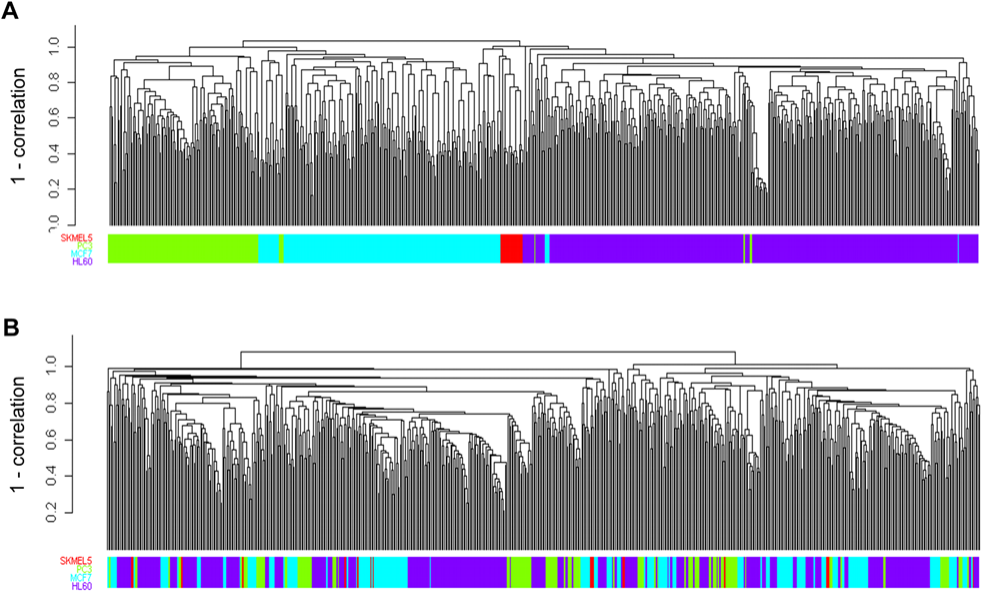
Hierarchical clustering of CMap instances less dominated by cell-type when clustering is based on gene-set/tag enrichment scores. Dendrograms are hierarchical clustering of CMap instances based on gene expression (A) and tag enrichment score (B). Colors in color bar below dendogram respectively represent the cell lines SKMEL5 (red), PC3 (green), MCF7 (blue), and HL60 (purple). For each instance top-300 genes or tags with the top-300 expression log-ratios or ES scores were selected for clustering based on Pearson distance metric and average linkage.

As a first comparison between the IGA and GSA, we separately hierarchically clustered the two (300x671) matrices tCMap1 (S1 Table, http://figshare.com/download/file/2258031), representing IGA, and tGSCMap1 (S2 Table, http://figshare.com/download/file/2258032), representing GSA, using a Pearson distance metric and average-linkage and examined the properties of the two resulting 671-branch dendograms as cell-type classifiers. Under visual inspection the tCMap1 dendrogram was overwhelmingly dominated by cell type (Fig 2A) whereas the tGSCMap1 dendogram was not (Fig 2B). Quantitatively, F-scores (Materials and methods) for the tCMap1 dendogram indicated that it provided a close to perfect classification for the four cell types (Table 1, permutation *p*-value < 0.01). In contrast, the tGSCMap dendogram was a poor (but fair for HL60) classifier for cell types. A similar result was found in a Principle Component Analysis on the full CMap dataset (S3 Fig). These results implied GSA results had a significantly better chance than IGA of not being masked by cell-type dependence.

**Table 1:** Cell-type effects are eliminated in hierarchical clustering based gene-set enrichment.

Cell type	F		Permutation *p*-value	
	Gene	Gene-set	Gene	Gene-set
MCF7	0.92	0.33	< 0.01	0.83
HL60	0.99	0.59	< 0.01	0.01
PC3	0.97	0.31	< 0.01	0.48
SKMEL5	1.00	0.30	< 0.01	0.09

Cluster evaluation by F score was computed for cell-type classification of hierarchical clustering based on individual genes and on gene-based enrichment (see materials and methods). Permutation *p*-value was calculated for the F scores by 100 random permutations of cell-type labels.

### Testing drug responses in IGA and GSA

#### GSA had clearer and more varied drug response than IGA

**Fig 3:**
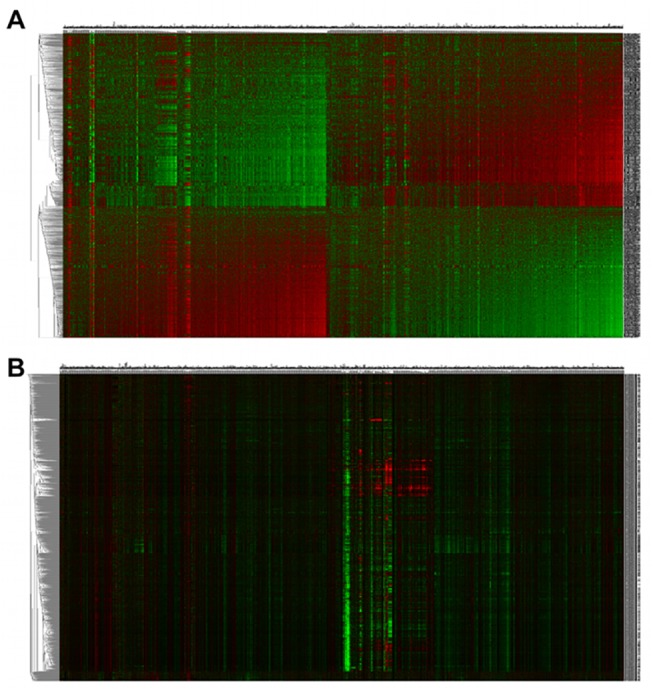
Two-way hierarchical clustering heatmep of 1309 CMap perturbagens shows higher contrast when clustering is based on gene-set/tag enrichment scores. Two-way hierarchical clustering heatmaps were generated based on Pearson distance metric and average linkage using, for each CMap perturbagen: (A) normalized enrichment scores (NESs) of 4,884 tags from MSigDB, and (B) log-ratios for expression levels of the top-4884 high-variance genes. Color code: on red, positive NES or log-ratio; green, black, NES or log ratio ~0; green, negative NES or log-ratio.

We separately two-way hierarchically clustered the two (4,884x1,309) matrices IGCMd (S3 Table, http://figshare.com/download/file/2258034) for IGA and GSCMd (S4 Table, http://figshare.com/download/file/2258033) for GSA using Pearson distance metric and average-linkage (Fig 3). All computations were carried out over two days on a personal computer with an Intel(R) dual core Quad CPU, 2.40 GHz processor with a 8GB RAM. While the vast majority of tags responded to the drugs as being either positively or negatively enriched (Fig 3A), the vast majority of high-variance genes were neither up-regulated nor down-regulated with respect to the drugs (Fig 3B).

#### GSA gave a better drug classifier than IGA

**Fig 4:**
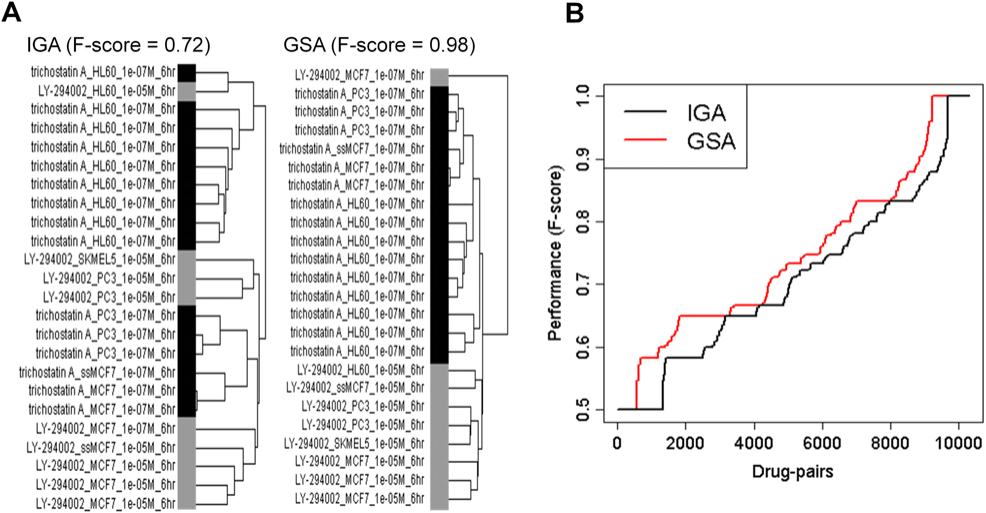
Separation of two drugs among all instances involving the drug pair is done better using gene-set/tag enrichment scores. Quality of separation is determined by F-scores for hierarchical clusters, constructed using tag enrichment scores and log-ratios for gene expressions, respectively, of all instances involving the drug pair. (A) Two clusters for the drug-pair valproic acid and trichostatin A; cluster based on gene expression, cluster on left, and on tag, cluster on right. Two-color bar indicates drug classification. (B) Ranking by F-scores of ~20,000 drug-pairs from the CMap development batch on HG-U133A platform involving 407 drugs and 674 chips; black, gene expression and red, tag.

In CMap a drug typically is represented by several instances. For example, the pairs of drugs, trichostatin and LY-294002, respectively occur in 15 and 9 instances, each instance represented by a vector of 4,884 ESs (in GSCMap) or 22,283 intensity log-ratios (in CMap). We separately hierarchically clustered the two sets of combined 24 instances. Viewed as classifiers of the two drugs, the GSA cluster had a F-score of 0.98, and the IGA cluster, 0.72 (Fig 4A). The superiority of GSA over IGA in its ability to tell one drug from another happened to be a general feature. We repeated the above comparison for all the 20,736 drug-pairs with multiple instances in CMap1 and in GSCMap1 and found that the (drug classification) F-score for GSA was about 0.036 higher then IGA over an average of 0.75 (Fig 4B, two-sample Kolmogorov-Smirnov test: *p*-value < 2.2e-16).

#### GSA and IGA responded similarly to chemical properties of CMap drugs

The F-scores of clusters, constructed through GSA (using ESs from GSCMd) and IGA (using gene expression log-ratios from IGCMd), of drugs classified according to their anatomical, chemical, therapeutic, pharmacological (Anatomical Therapeutic Chemical (ATC) classification system, World Health Organization, http://www.whocc.no/) and structural (PubChem Structure Database [44]) properties (Material and Methods) were indistinguishable (S4 Fig).

#### Genomic signatures of same-target drug pairs had higher correlation in GSA than in IGA

**Fig 5:**
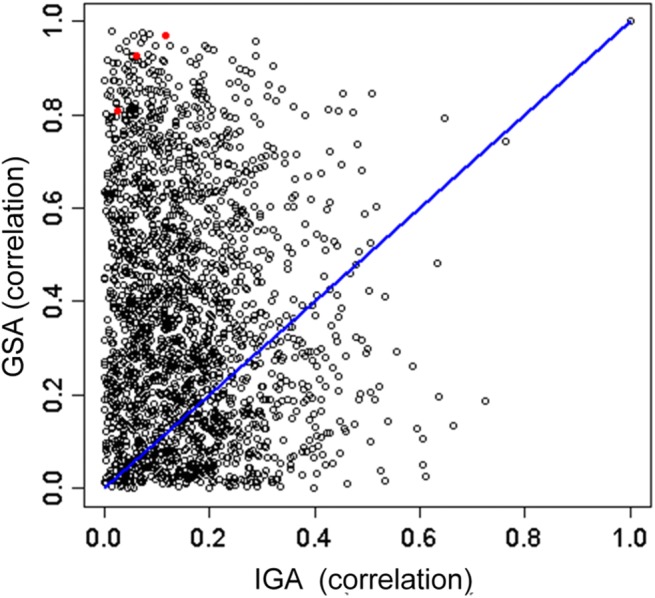
Same-target drug-pairs correlate better when evaluated by gene-set/tag enrichment scores. Figure plots correlation of same-target drug pair evaluated by tag enrichment score (ES) versus that evaluated by gene expression. Drug targets were those given by TTD database. In the tag approach, each drug, or CMap perturbagen, was represented by the ESs of 4884 MSigDB tags. In the gene expression case, each drug was represented by the set of top-4884 high variance genes. The three red dots are from the three pairs formed by the three drugs, vorinostat, valproic acid, and trichostatin A, all targeting the histone deacetylase (HDAC) protein.

We expect the genomic signatures of drugs sharing a target to be more similar than drugs that do not. Information on drug targets were obtained from the Therapeutic Target Database (TTD) [41] (Material and Methods). The same-target drug-pairs correlated much better under GSA (ESs from GSMCd) than IGA (gene expression log-ratios from IGMCd) (Fig 5). An outstanding case was the triplet vorinostat, valproic acid, and trichostatin A that targets the histone deacetylase (HDAC) protein. The three pair-wise correlations for the triplet ranged from 0.8 to 1.0 in GSA and from 0.05 to 0.15 in IGA. Averaged over all 5,034 pairs involving 639 drugs, the mean of GSA correlation was 0.35 (S.D. = 0.27) and the mean of IGA correlation was 0.18 (S.D. = 0.15) (two-sample *t*-test, *p*-value < 2.2e-16).

### Validation of GSLHC and novel HDAC inhibitors

**Fig 6:**
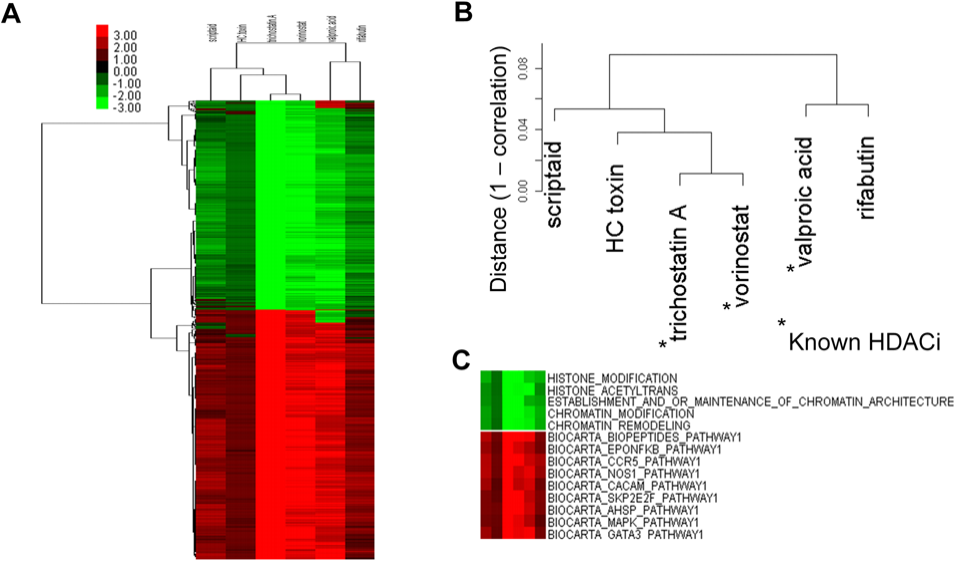
GSLHC finds novel HDAC inhibitors. The three know HDAC inhibitors valproic acid, trichostatin A, and vorinostat, are all significantly enriched by 597 tags with permutation p< 0.005; these 597 tags were used in a new heatmap in the GSLHC protocol. (A) A sub-heatmap including the three HDAC inhibitors and all neighbors with correlation > 0.9. (B) Detail of the drug cluster associated with the sub-heatmap. The two drugs rifabutin and scriptaid in the cluster, not previously known as HDAC inhibitors, has literature support as having inhibition functions on HDAC proteins. (C) Detail of the tag cluster with the sub-heatmap shows several functions known to be related to HDAC inhibitor activities.

There are 106 active compounds in the CMap database that are poorly studied, and GSLHC was developed as an application on GSCMap to discover drug partners of known therapeutic properties for the compounds. We tested the GSLHC by giving it a set of tags common to and significantly enriched in the functional profiles of three histone deacetylase (HDAC) inhibitors–vorinostat (also known as suberoylanilide hydroxamic acid or SAHA), valproic acid, and trichostatin A–and see if it can recover them from GSCMap. The three HDAC inhibitors were chosen because they have been fully studied [45–47]. A set of 597 tags significantly enriched with permutation *p*< 0.005 were selected for the test (Material and Methods). The selected tags had functions related to HDAC inhibitor activities. For example, among the down-regulated functions were histone acetylating, histone and chromatin modification, and maintenance of chromatin structures (Fig 6C). The test was successful; the triplet was among the six recovered drugs (Fig 6A and 6B). The three extras are not known as HDAC inhibitors but two of the three, scriptaid and HC toxin, have been reported to have HDAC inhibition activities [48, 49].

### Sample applications of GSLHC to characterization of active compounds

#### A novel cyclin-dependent kinase inhibitor (CDKi)

**Fig 7:**
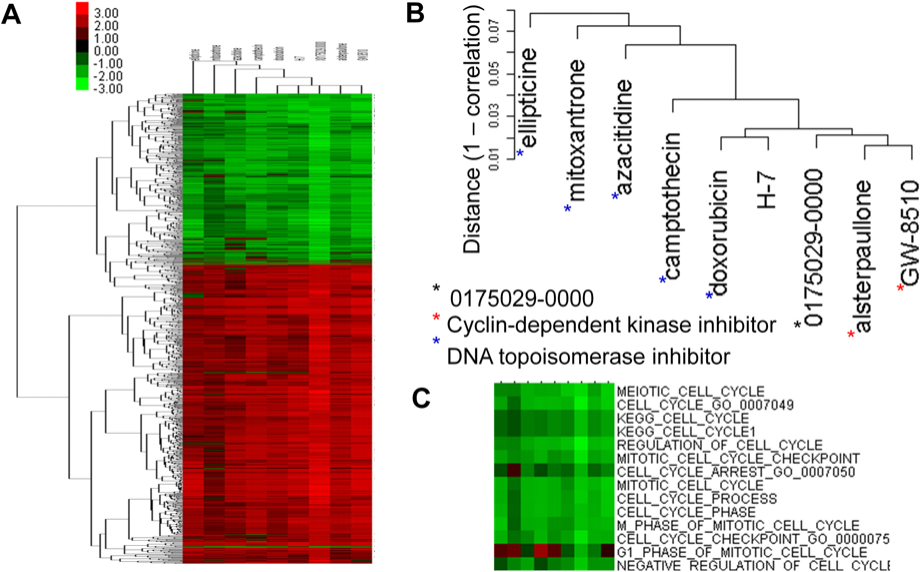
GSLHC identifies 0175029–0000 as a novel cyclin-dependent kinase inhibitor (CDKi). (A) A correlation > 0.9 sub-heatmap including the compound 0175029–0000 of unknown function from a GSLHC-generated heatmap based on the ES of 1080 tags significantly enriched in 0175029–0000 with permutation p< 0.005. (B) Detail of the drug cluster associated with the sub-heatmap. According to the TTD database, GW-8510, alsterpaullone, and H-7 (red asterisk) CDK inhibitors, and doxorubicin, camptothecin, azacitidine, mitoxantrone, and ellipticine (blue asterisk) are DNA topoisomerase inhibitors. All have anti-tumor activities. (C) Detail of the tag cluster with the sub-heatmap shows functions known to be related to the inhibition activities of cell cycle.

The compound 0175029–0000 is among molecules in CMap known to be active in certain biological roles [11] but poorly studied in literature. Its ES profile had 1,080 significantly enriched tags with permutation *p*< 0.005. Our GSLHC search showed it to be closely associated with three CDKi’s with correlation coefficient (CE) > 0.97 and five DNA topoisomerases with CE > 0.92 (Fig 7A and 7B). Biological functions negatively regulated by these drugs included those related to cell cycle and checkpoint on cell cycle (Fig 7C).

#### A novel antibiotic, anesthetic, and anti-inflammatory agent

**Fig 8:**
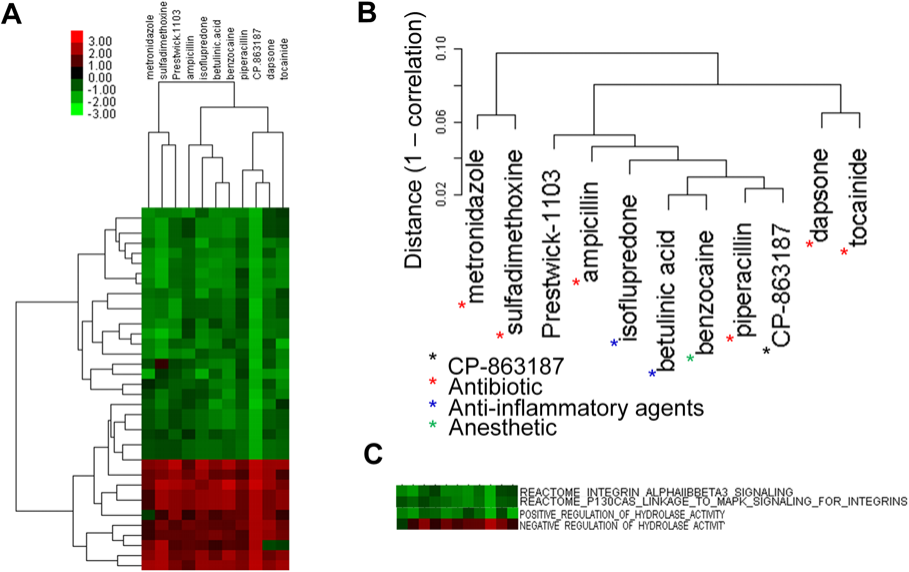
GSLHC identifies CP-863187 as a potential antibiotic. (A) A correlation > 0.9 sub-heatmap including the compound CP-863187 of unknown function from a GSLHC-generated heatmap based on the ES of 36 tags significantly enriched in CP-863187 with permutation p< 0.005. (B) Detail of the drug cluster associated with the sub-heatmap. According to TTD database, piperacillin, dapsone, tocainide, ampicillin, sulfadimethoxine, metronidazole (red asterisk) are antibiotics, betulinic acid and isoflupredone (blue asterisk) are anti-inflammatory agents, and benzocaine (green asterisk) is an anesthetic. (C) Detail of the tag cluster with the sub-heatmap shows functions known to block the formation of bacteria cell wall by inhibition of integrin signaling pathway.

The ES profile of compound CP-863187 had 36 significantly enriched tags with permutation p< 0.005. Our GSLHC search showed it to be closely associated with an antibiotic (piperacilin; CE > 0.98), an anesthetic (benzocaine), an anti-inflammatory agents (betunlinic acid; CE > 0.97), as well as with another anti-inflammatory agent (CE > 0.96) and five other antibiotics (CE > 0.90) (Fig 8A and 8B). Biological functions affected by drugs associated with CP-863187 included negative regulation of integrin signalling pathway and hydrolases (Fig 8C).

### Summary of drug discovery by GSLHC (Table 2)

Eighteen previously uncharacterized compounds in CMap, including 0175029–0000 and CP-863187, were discovered by GSLHC to have closely associated drug partners (in CMap), putative targets, and therapeutic indications (Table 2; detail in S5–S20 Figs). Among the discoveries, eight compounds: tyrphostin AG-825, 5248896, 0175029–0000, H-7, U0125, STOCK1N-35215, 0297417-0002B, and F0447-0125, were identified as having potential anti-tumor activities. Depending on their closest putative drug partners, their molecular mechanisms differ. Camptothecin, irinotecan, and betulinic acid, with closest partners tyrphostin AG-825, U0125, and CP-944629, respectively, were predicted to block DNA transcription by inhibiting DNA topoisomerase activities. The compounds 0175029–0000 and H-7, with closest partner GW-8510, were predicted to be cyclin-dependent kinase inhibitors. Compounds predicted to have therapeutic activities on non-cancer diseases include 5186324 (closest partner neostigmine bromide and therapeutic activity on myasthenia gravis) and Prestwick-692 (closest partner isoflupredone and therapeutic activity on rheumatoid arthritis).

**Table 2:** Putative molecular target and pharmacology obtained from application of GSLHC on CMap perturbagens without known indication.

Test drug (+ anti-tumor)	Cor.	Partner drug	Partner drug targets	Indications
5186324	0.99	Neostigmine bromide	Acetylcholinesterase inhibitor	Myasthenia gravis
DL-PPMP	0.99	Indoprofen	Cyclooxygenase-1 inhibitor	Non-steroidal anti-inflammatory drug
Prestwick-692	0.99	Isoflupredone	Glucocorticoid receptor agonist	Rheumatoid arthritis
tyrphostin AG-825^+^	0.995	Camptothecin	DNA topoisomerase I inhibitor	Cancer
	0.990	GW-8510	Cyclin-dependent kinase 2 inhibitor	Cancer
	0.990	Doxorubicin	DNA topoisomerase II inhibitor	Cancer
	0.975	duanorubicin	DNA topoisomerase II inhibitor	Leukemia, cancer
	0.970	Irinotecan	DNA topoisomerase I inhibitor	Colorectal Cancer
	0.96	Mitoxantrone	Human epidermal growth factor receptor-2/neu inhibitor	Acute myeloid leukemia, metastatic breast cancer
	0.96	alsterpaullone	Glycogen synthase kinase 3 inibitor	Cancer, type II diabetes
5248896^+^	0.98	tyrphostin AG-825	Human epidermal growth factor receptor-2/neu inhibitor	Myeloid leukemia
0175029–0000^+^	0.98	GW-8510	Cyclin-dependent kinase 2 inhibitor	Cancer
CP-863187	0.98	Piperacillin	Sodium channel blocker	Anesthetic
H-7^+^	0.98	GW-8510	Cyclin-dependent kinase 2 inhibitor	Cancer
	0.98	Doxorubicin	anthracycline antibiotic	Cancer
Prestwick-1103	0.98	Pentoxifylline	Tumor necrosis factor antibody	Intermittent claudication, vascular dementia
U0125^+^	0.98	Irinotecan	DNA topoisomerase I inhibitor	Colorectal Cancer
5109870	0.97	Phenoxybenzamine	Alpha adrenergic receptor antagonist	Hypertension, hypoplastic left heart syndrome
MG-132	0.97	MG-262	Proteasome Inhibitor	—-
PHA-00851261E	0.97	Amrinone (inamrinone)	CGMP-inhibited 3',5'-cyclic phosphodiesterase	Congestive heart failure
STOCK1N-35215^+^	0.97	MS-275	Histone deacetylase inhibitor	Hodgkin's lymphoma (phase II trial)
0297417-0002B^+^	0.95	8-azaguanine	Purine nucleoside phosphorylase Inhibitor	Acute leukemia
F0447-0125^+^	0.95	Lomustine	DNA Inhibitor	Brain tumours, Hodgkin's lymphoma
W-13	0.95	Fludrocortisones	Mineralocorticoid receptor agonist	Addison's disease, cerebral saltwasting syndrome
CP-944629	0.92	Betulinic acid	DNA polymerase beta inhibitor	Melanoma (in deve-lopment)

Partner drug of a perturbagen (test drug in first column) was given by GSLHC. Partner drug target and indication were those associated with partner drug as given by the TTD database. Target of the test drug may not be that of the partner drug. Perturbagens found to be anti-tumouric are marked by the ^+^ symbol.

### GSLHC website (http://cloudr.ncu.edu.tw/gslhc/)

This website contains 1,857 local hierarchical clusters accessible by querying 555 of the 1,309 drugs and small molecules listed in CMap v2.0. The other CMap drugs do not yield local hierarchical clusters that meet the criteria permutation p-values not greater than 0.01 and Pearson correlation not less than 0.90. The full dataset of NES values (http://figshare.com/download/file/2288071) and permutation p-values (http://figshare.com/download/file/2288072) for generating the hierarchical cluster results shown in GSLHC website can be downloaded and replicated in the local computer.

## Discussion and Summary

We used CMap as a vehicle for the demonstration that GSA is a better way than IGA in utilizing genome-wide gene expression. Because this would involve repeated and massive application of CMap, we constructed a local extended version of CMap. The local CMap was stored and computation using it were conducted on a personal computer equipped with Intel(R) dual core Quad CPU, 2.40 GHz processor with a 8GB RAM. Advantages of the local program over the remote CMap include: (i) No reliance on the Internet and the ensuing network connection time saved; (ii) Length of the list of querying gene not limited to 1000; (iii) Capability for batch mode operation. Extensive tests conducted on the local version confirmed its accuracy, and verified that in single mode its running speed is comparable to the remote CMap (S1 Fig).

We implemented a GSA-based application of CMap by constructing GSCMap, an analog of CMap where gene-based genomic profiles of instances in CMap are replaced by tag-based functional profiles.

Hierarchical clustering based on gene expression has been an important tool in genomic technology. We showed that IGA-based hierarchical clustering of the CMap (the matrix) was dominated by cell-types, a dominance absent in the GSA-based GSCMap (Fig 2). This notion was strengthened by our quantitative measure, using F-scores, of the clusters as classifies of cell types. We confirmed a previous report that CMap was an excellent classifier of cell types, a result that imposes strong constraints of it being a good classifier of drug effects. In contrast, our F-score analysis showed GSCMap to be a poor classifier of cell types (Table 2). It is biologically reasonable that drug sensitivity varies with different cell types [50]. In a method that studies drug effects using a database such as CMap, the question is whether the method can pick out drug-induced signals over the background of cell type-specific signatures. For instance, the clustering of instances of the two drugs trichostatin and LY-294002 shows that under IGA, signatures associated with the cell line HL60 dominate over drug effects, whereas under GSA, drug-specific signals dominates over cell type-specific signatures (Fig 4A). This suggests that GSA provides a better means than IGA for focusing on drug effects, or “shared factors” [22], that are common to different cell types.

Having demonstrated that GSCMap has far weaker cell-type dependence than CMap, we conducted three tests to show the former had more discriminating responses to drug properties than the latter. The first test (using the 4,884x1,309 matrices GSCMd and IGCMd) showed tag response to drugs in GSCMap exhibited a much wider range then gene expression response to drugs in CMap (Fig 3). A second test showed that GSCMap clustered same-drug instances consistently better than CMap (Fig 4). A third test showed that the genomic profiles of a pair of drugs having the same target had higher correlation in GSCMap than in CMap (Fig 5). Our assumption for the third test is that same-target drugs are designed to have similar indication. Based on this assumption, the result of the test—the GSEA-based and IGA-based correlations have a two-sample *t*-test *p*-value of < 2.2e-16—suggests GSCMap much better connects drugs with similar indication. The case of the three HDAC inhibitors–vorinostat, valproic acid, and trichostatin A–brings home this point (red dots in Fig 5; admittedly this represents an extreme case). In GSEA the three pairwise correlations among the three drugs have a mean value of 0.90 (SD = 0.082), and in GSA the mean correlation is 0.077 (SD = 0.055). The *t*-test *p*-value for the two sets is 0.00301. Thus, in the IGA mode, if a query (a genomic profile or a gene set) matches (i.e., has a high IGA enrichment score) with one of the three HDAC inhibitors, it will not match either of the other two. In contrast, in the GSEA mode, a query will either match all three HDAC inhibitors or not match any.

Similar correlation-based analysis applied to drug-pairs having structural similarities at the chemical level or therapeutic indications at the clinical level did not exhibit any different between GSCMap and CMap (S4 Fig). This is not surprising, since global genomic signatures do not generally bear any direct relation to chemical structures of the drug and the target. Chemical compatibility between drug and target is a crucial consideration in drug design, especially when the purpose is to regulate a specific target that has a central role in a biological pathway. CMap (hence GSCMap) was not constructed to address the question of chemical compatibility. CMap focuses on the effects of a drug as manifested in changes it causes in the genomic profile, but makes no assumption on how those changes were brought about. This implies that in Table 2, the test drug may not share the target of the partner drug.

GSLHC was designed to discover, through GSCMap, functional links among drugs in CMap. The principle of the method, local hierarchical clustering, is generally applicable to any large list that may or may not represent drug effects. We validated GSLHC by using three known HDAC inhibitors as bait and saw that they were recovered as part of a tight cluster returned by GSLHC (red dots in Fig 5). The cluster also included three drugs, scriptaid, HC toxin, and rufabutin, not previously known as HDAC inhibitors. GSLHC showed all three as having significant correlation with biological functions relating to switching histone modification and destroying chromatin maintenance (Fig 6); scriptaid and HC toxin have been reported to inhibit HDAC proteins [48, 49], and rifabutin is primarily used in the treatment of tuberculosis. We regard all three as potential novel HDAC inhibitors.

Of the 106 uncharacterized compounds in the CMap dataset, GSLHC found drug partners of known indications for 18 (Table 2), 8 of which, tyrphostin AG-825, 0175029–0000, H-7, U0125, STOCK1N-35215, 0297417-0002B, F0447-0125, and CP-944629 were inferred to have anti-tumor activities. In each case we found significantly correlations between the compound with newly inferred indication and biological functions related to that indication (Figs 7 and 8, and S5–S20 Figs). As mentioned, these predictions do not make any statement about drug targets.

The compound 0175029–0000 was shown to be closely associated with three CDKi’s–GW-8510 [51–58], alsterpaullone [51–58], H-7 [51–58]–and five DNA topoisomerases–doxorubicin [51–58], camptothecin [51–58], azacitidine [51–58], mitoxantrone [51–58], and ellipticine [51–58] (Fig 7), and was inferred as a putative CDKi/DNA topoisomerases, all of which have been reported to have anti-tumour activities [51–58] and significantly expressed biological functions that negatively regulate cell cycle and checkpoint on cell cycle (Fig 7C).

The compound CP-863187 was shown to be closely associated with an antibiotic (piperacilin), an anesthetic (benzocaine), and an anti-inflammatory agent (betunlinic) (Fig 8), and to significantly express negative regulation of integrin signaling and hydrolases (Fig 8C). There are studies suggesting that antibiotics may have inflammatory and anesthetic properties [59, 60]. The source of the shared properties may be that as a signal transductors, integrins are involved in activities on cell membranes and cell-cell interactions. Hydrolases are ubiquitous and play important roles among bacteria including digesting the murein of bacteria [61], acting as a pacemaker for cell wall growth [62], and splitting the septum during cell division [63].

Despite its apparent success, the GSLHC approach has its own limitations. Statistical concerns regarding the neutrality of GSEA has been raised [64, 65] (and replied [64, 65]). There is not a perfect method for extracting hypothesis-free information from something as rich as a modern set of genome-wide gene expression data. The several tests shown in this work does show that for practical purposes, GSA, including GSEA and two algorithms derived from it, PAGE and GAGE, is superior to IGA. Of the 106 unknown compounds in CMap (version 2.0), we only found drug partners for 18. That we failed to do the same for the other 88 compounds have many possible reasons: a weakness of GSLHC; the tags in MSigDB is not sufficiently comprehensive; the sets of compounds presently included in MCap is too restrictive. Improvements on all three fronts are possible, even expected. Already in its current form, we expect the GSLHC approach to be more widely applicable to many areas other than what was demonstrated here. To name a few: repurposed drug discovery based on functional-profile characterization of phenotypes, function-based diagnosis and classification of complex diseases, and prognosis on advance-stage patients after chemotherapy treatment.

A sequel to CMap, the LINCS L1000 dataset consisting of over 1.4M gene-expression profiles collected from human cells treated with chemical compounds, was recently constructed and made available online (http://support.lincscloud.org/hc/en-us) by The Broad Institute. In L1000 each profile is a 1000-gene representation of a gene-expression profiling assay based on the direct measurement of the transcriptome. A GSA as carried out in the present paper will not be suitable for L1000 gene-sets. However, it will be interesting to investigate the cell-type dependence of the LINCS data.

## Supporting Information

S1 Fig The local program reproduces results of CMap server. (A) The local program (blue) tracks results given by CMap for permutation p-value (solid lines), with small deviations when drug list is less than 30, and enrichment score (dash lines). (B) Run times for the local program and CMap are comparable, with the former slightly faster when size of probe set is less than 700, and slight slower otherwise.
(TIF)

S2 Fig Most of replicates treating with the same perturbagen show low reproducibility on the top-1000 differentially expressed genes (DEGs) across all CMAP datasets. The reproducibility between two treatments (blue: the same perturbagen; red: two different perturbagens) is defined by the frequency of number of the overlapping genes verse the number of 1000 DEGs.
(TIF)

S3 Fig Principle component analysis of full C-MAP dataset. The first two components, together accounting for 21.7% of the total weight, show a clear separation of data from the HC60 (black circle) and PC3 (green cross) cell lines.
(TIF)

S4 Fig Performance test (F-score) showed that no difference between gene and tag clusters by Anatomical Therapeutic Chemical (ATC) classification system and PubChem structure database. (A) In PubChem database, we use chemical structure clustering tool to cluster compounds based on the structure (fingerprint) similarity using the Single Linkage algorithm; number of cluster decreases with cluster size. Both results indicated that F-score increases with decreasing class size. (B) In ATC system, drugs are classified into groups at 4 different levels–from general anatomical groups to detail chemical/therapeutic/pharmacological subgroups.
(TIF)

S5 Fig GSLHC identified the compound 5186324 as a novel acetylcholinesterase inhibitor. (A) A correlation > 0.9 sub-heatmap including the compound 5186324 of unknown function from a GSLHC-generated heatmap based on tags significantly in 5186324 enriched with permutation p< 0.005. (B) Detail of the dendrogram showing 5186324 (marked by black asterisk) with its partner drugs.
(TIF)

S6 Fig GSLHC identified the compound DL-PPMP as a novel cyclooxygenase-1 inhibitor. (A) A correlation > 0.9 sub-heatmap including the compound DL-PPMP of unknown function from a GSLHC-generated heatmap based on tags significantly enriched in DL-PPMP with permutation p< 0.005. (B) Detail of the dendrogram showing DL-PPMP (marked by black asterisk) with its partner drugs.
(TIF)

S7 Fig GSLHC identified the compound Prestwick-692 as a novel glucocorticoid receptor agonist. (A) A correlation > 0.9 sub-heatmap including the compound Prestwick-692 of unknown function from a GSLHC-generated heatmap based on tags significantly enriched in Prestwick-692 with permutation p< 0.005. (B) Detail of the dendrogram showing Prestwick-692 (marked by black asterisk) with its partner drugs.
(TIF)

S8 Fig GSLHC identified the compound tyrphostin AG-825 as a novel DNA topoisomerase I inhibitor. (A) A correlation > 0.9 sub-heatmap including the compound tyrphostin AG-825 of unknown function from a GSLHC-generated heatmap based on tags significantly enriched in tyrphostin AG-825 with permutation p< 0.005. (B) Detail of the dendrogram showing tyrphostin AG-825 (marked by black asterisk) with its partner drugs.
(TIF)

S9 Fig GSLHC identified the compound 5248896 as a novel human epidermal growth factor receptor (HER)-2/neu inhibitor. (A) A correlation > 0.9 sub-heatmap including the compound 5248896 of unknown function from a GSLHC-generated heatmap based on tags significantly enriched in 5248896 with permutation p< 0.005. (B) Detail of the dendrogram showing 5248896 (marked by black asterisk) with its partner drugs.
(TIF)

S10 Fig GSLHC identified the compound H-7 as a novel Cyclin-dependent kinase 2 Inhibitor. (A) A correlation > 0.9 sub-heatmap including the compound H-7 of unknown function from a GSLHC-generated heatmap based on tags significantly enriched in H-7 with permutation p< 0.005. (B) Detail of the dendrogram showing H-7 (marked by black asterisk) with its partner drugs.
(TIF)

S11 Fig GSLHC identified the compound Prestwick-1103 as a novel Tumor necrosis factor antibody. (A) A correlation > 0.9 sub-heatmap including the compound Prestwick-1103 of unknown function from a GSLHC-generated heatmap based on tags significantly enriched in Prestwick-1103 with permutation p< 0.005. (B) Detail of the dendrogram showing Prestwick-1103 (marked by black asterisk) with its partner drugs.
(TIF)

S12 Fig GSLHC identified the compound U0125 as a novel DNA topoisomerase I inhibitor. (A) A correlation > 0.9 sub-heatmap including the compound U0125 of unknown function from a GSLHC-generated heatmap based on tags significantly enriched in U0125 with permutation p< 0.005. (B) Detail of the dendrogram showing U0125 (marked by black asterisk) with its partner drugs.
(TIF)

S13 Fig GSLHC identified the compound 5109870 as a novel Alpha adrenergic receptor antagonist. (A) A correlation > 0.9 sub-heatmap including the compound 5109870 of unknown function from a GSLHC-generated heatmap based on tags significantly enriched in 5109870 with permutation p< 0.005. (B) Detail of the dendrogram showing 5109870 (marked by black asterisk) with its partner drugs.
(TIF)

S14 Fig GSLHC identified the compound MG-132 as a novel Proteasome Inhibitor. (A) A correlation > 0.9 sub-heatmap including the compound MG-132 of unknown function from a GSLHC-generated heatmap based on tags significantly enriched in MG-132 with permutation p< 0.005. (B) Detail of the dendrogram showing MG-132 (marked by black asterisk) with its partner drugs.
(TIF)

S15 Fig GSLHC identified the compound PHA-00851261E as a novel CGMP-inhibited 3',5'-cyclic phosphodiesterase. (A) A correlation > 0.9 sub-heatmap including the compound PHA-00851261E of unknown function from a GSLHC-generated heatmap based on tags significantly enriched in PHA-00851261E with permutation p< 0.005. (B) Detail of the dendrogram showing PHA-00851261E (marked by black asterisk) with its partner drugs.
(TIF)

S16 Fig GSLHC identified the compound STOCK1N-35215 as a novel Histone deacetylase inhibitor. (A) A correlation > 0.9 sub-heatmap including the compound STOCK1N-35215 of unknown function from a GSLHC-generated heatmap based on tags significantly enriched in STOCK1N-35215 with permutation p< 0.005. (B) Detail of the dendrogram showing STOCK1N-35215 (marked by black asterisk) with its partner drugs.
(TIF)

S17 Fig GSLHC identified the compound 0297417-0002B as a novel Purine nucleoside phosphorylase Inhibitor. (A) A correlation > 0.9 sub-heatmap including the compound 0297417-0002B of unknown function from a GSLHC-generated heatmap based on tags significantly enriched in 0297417-0002B with permutation p< 0.005. (B) Detail of the dendrogram showing 0297417-0002B (marked by black asterisk) with its partner drugs.
(TIF)

S18 Fig GSLHC identified the compound F0447-0125 as a novel DNA Inhibitor. (A) A correlation > 0.9 sub-heatmap including the compound F0447-0125 of unknown function from a GSLHC-generated heatmap based on tags significantly enriched in F0447-0125 with permutation p< 0.005. (B) Detail of the dendrogram showing F0447-0125 (marked by black asterisk) with its partner drugs.
(TIF)

S19 Fig GSLHC identified the compound W-13 as a novel Mineralocorticoid receptor agonist. (A) A correlation > 0.9 sub-heatmap including the compound W-13 of unknown function from a GSLHC-generated heatmap based on tags significantly enriched in W-13 with permutation p< 0.005. (B) Detail of the dendrogram showing W-13 (marked by black asterisk) with its partner drugs.
(TIF)

S20 Fig GSLHC identified the compound CP-944629 as a novel DNA polymerase beta inhibitor. (A) A correlation > 0.9 sub-heatmap including the compound CP-944629 of unknown function from a GSLHC-generated heatmap based on tags significantly enriched in CP-944629 with permutation p< 0.005. (B) Detail of the dendrogram showing CP-944629 (marked by black asterisk) with its partner drugs.
(TIF)

S1 Table The 300x671 tCMap1 matrix used to construct the one-way cluster in Fig 2A. tCMap1 is the 300 by 671 similarity matrix of the 300 highest variance microarray probe-sets and the 671 instances in CMap v1.0 (http://figshare.com/download/file/2258031).
(XLSX)

S2 Table The 300x671 tGSCMap1 matrix used to construct the one-way cluster in Fig 2B. tGSCMap1 is the 300 by 671 similarity matrix of the 300 largest ES variance MSigDB tags and the 671 instances in CMap v1.0 (http://figshare.com/download/file/2258032).
(XLSX)

S3 Table The 4884x1309 IGCMd matrix used to construct the two-way cluster in Fig 3A. IGCMd is the 4884 by 1309 similarity matrix of the 4884 highest variance microarray probe-sets against the 1309 drugs/chemicals in CMap v2.0 (http://figshare.com/download/file/2258034).
(XLSX)

S4 Table The 4884x1309 GSCMd matrix used to construct the two-way cluster in Fig 3B. GSCMd is the 4884 by 1309 similarity matrix of the 4884 largest ES variance MSigDB tags against the 1309 drugs/chemicals in CMap v2.0 (http://figshare.com/download/file/2258033).
(XLSX)
