# Supplementary figures and images for: Gene-Set Local Hierarchical Clustering (GSLHC)—A Gene Set-Based Approach for Characterizing Bioactive Compounds in Terms of Biological Functional Groups
Source: PLoS One. 2015 Oct 16;10(10):e0139889. doi: 10.1371/journal.pone.0139889 (PMC4652590; doi:10.1371/journal.pone.0139889)

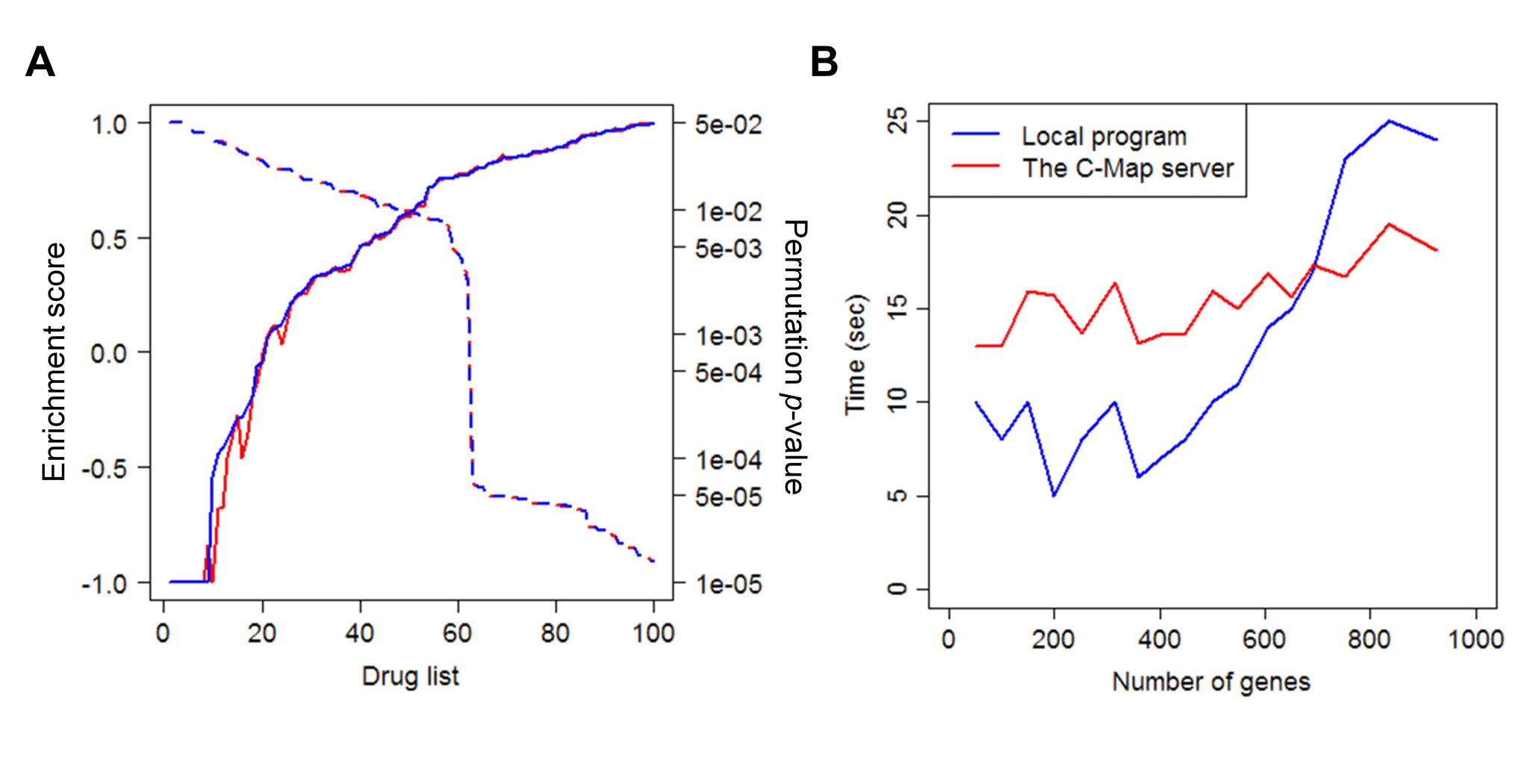

Supplement: S1 Fig — (A) The local program (blue) tracks results given by CMap for permutation p-value (solid lines), with small deviations when drug list is less than 30, and enrichment score (dash lines). (B) Run times for the local program and CMap are comparable, with the former slightly faster when size of probe set is less than 700, and slight slower otherwise. (TIF) [file pone.0139889.s001.tif]

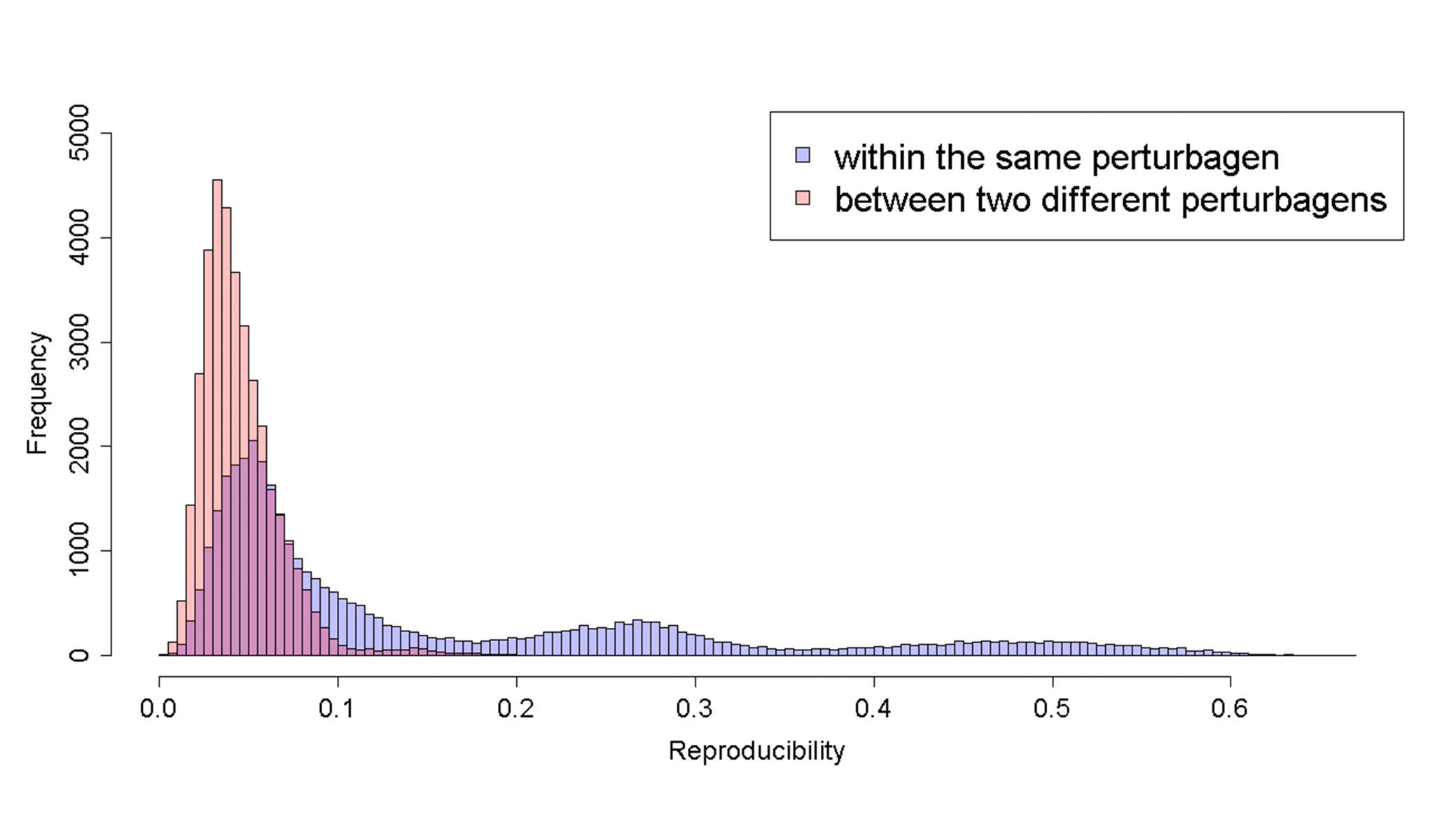

Supplement: S2 Fig — The reproducibility between two treatments (blue: the same perturbagen; red: two different perturbagens) is defined by the frequency of number of the overlapping genes verse the number of 1000 DEGs. (TIF) [file pone.0139889.s002.tif]

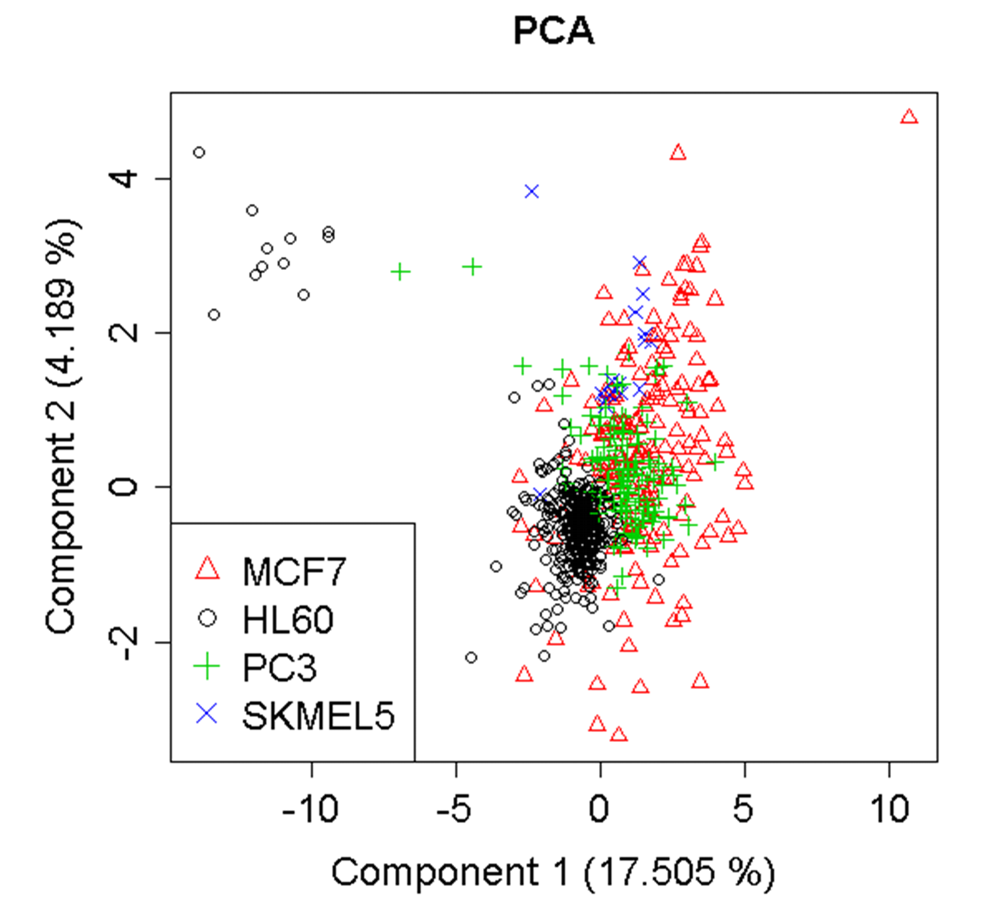

Supplement: S3 Fig — The first two components, together accounting for 21.7% of the total weight, show a clear separation of data from the HC60 (black circle) and PC3 (green cross) cell lines. (TIF) [file pone.0139889.s003.tif]

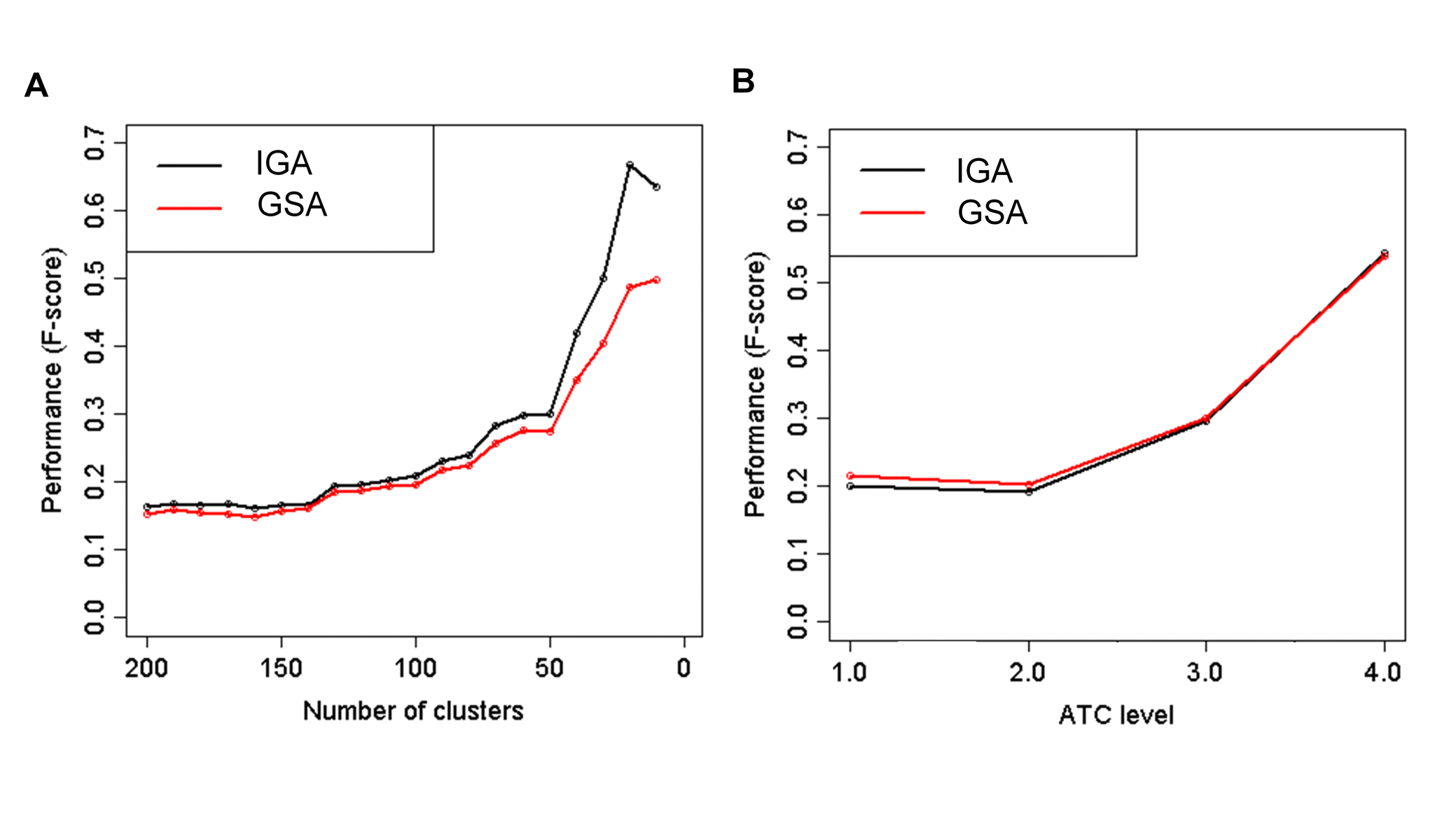

Supplement: S4 Fig — (A) In PubChem database, we use chemical structure clustering tool to cluster compounds based on the structure (fingerprint) similarity using the Single Linkage algorithm; number of cluster decreases with cluster size. Both results indicated that F-score increases with decreasing class size. (B) In ATC system, drugs are classified into groups at 4 different levels–from general anatomical groups to detail chemical/therapeutic/pharmacological subgroups. (TIF) [file pone.0139889.s004.tif]

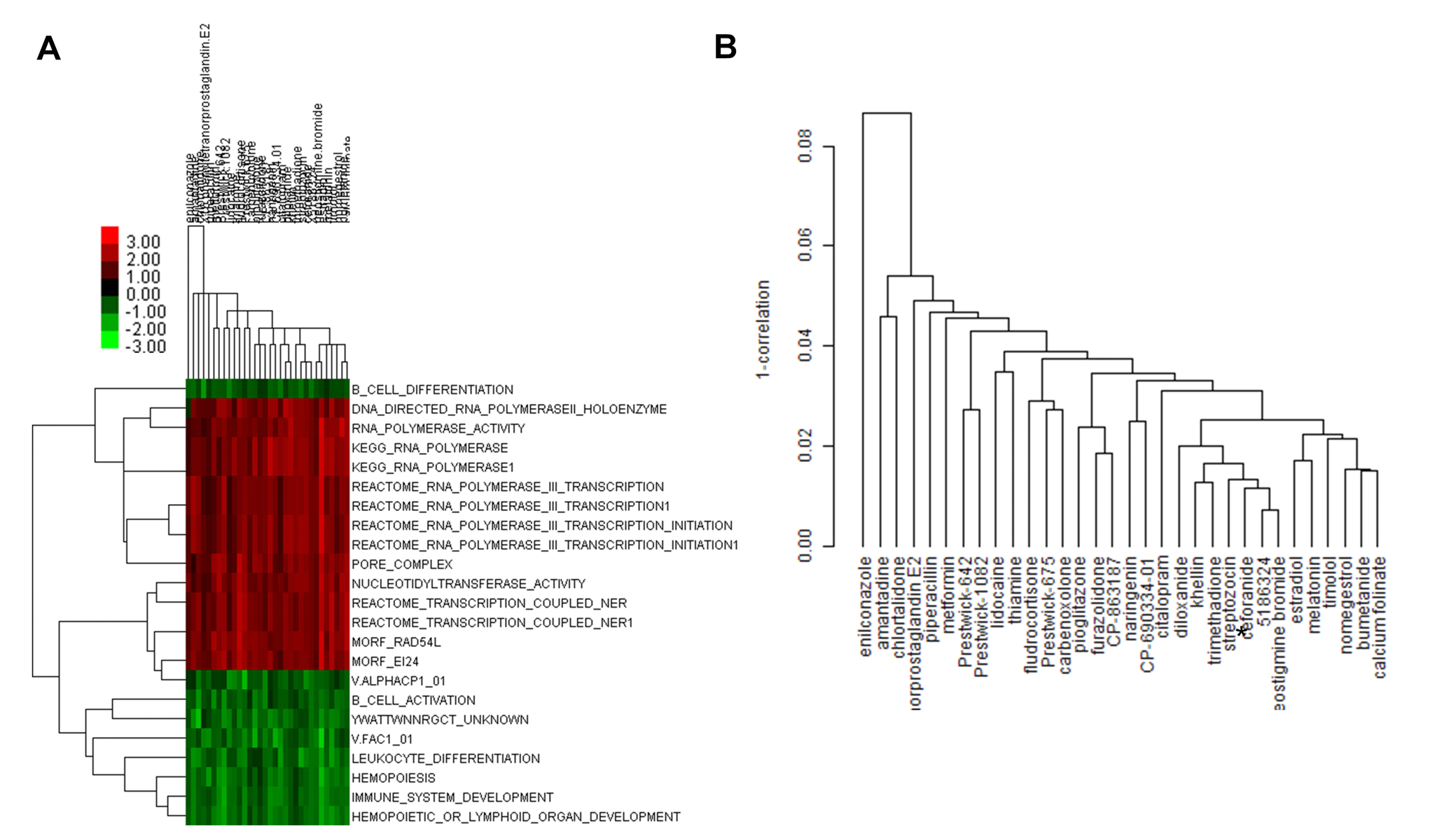

Supplement: S5 Fig — (A) A correlation > 0.9 sub-heatmap including the compound 5186324 of unknown function from a GSLHC-generated heatmap based on tags significantly in 5186324 enriched with permutation p< 0.005. (B) Detail of the dendrogram showing 5186324 (marked by black asterisk) with its partner drugs. (TIF) [file pone.0139889.s005.tif]

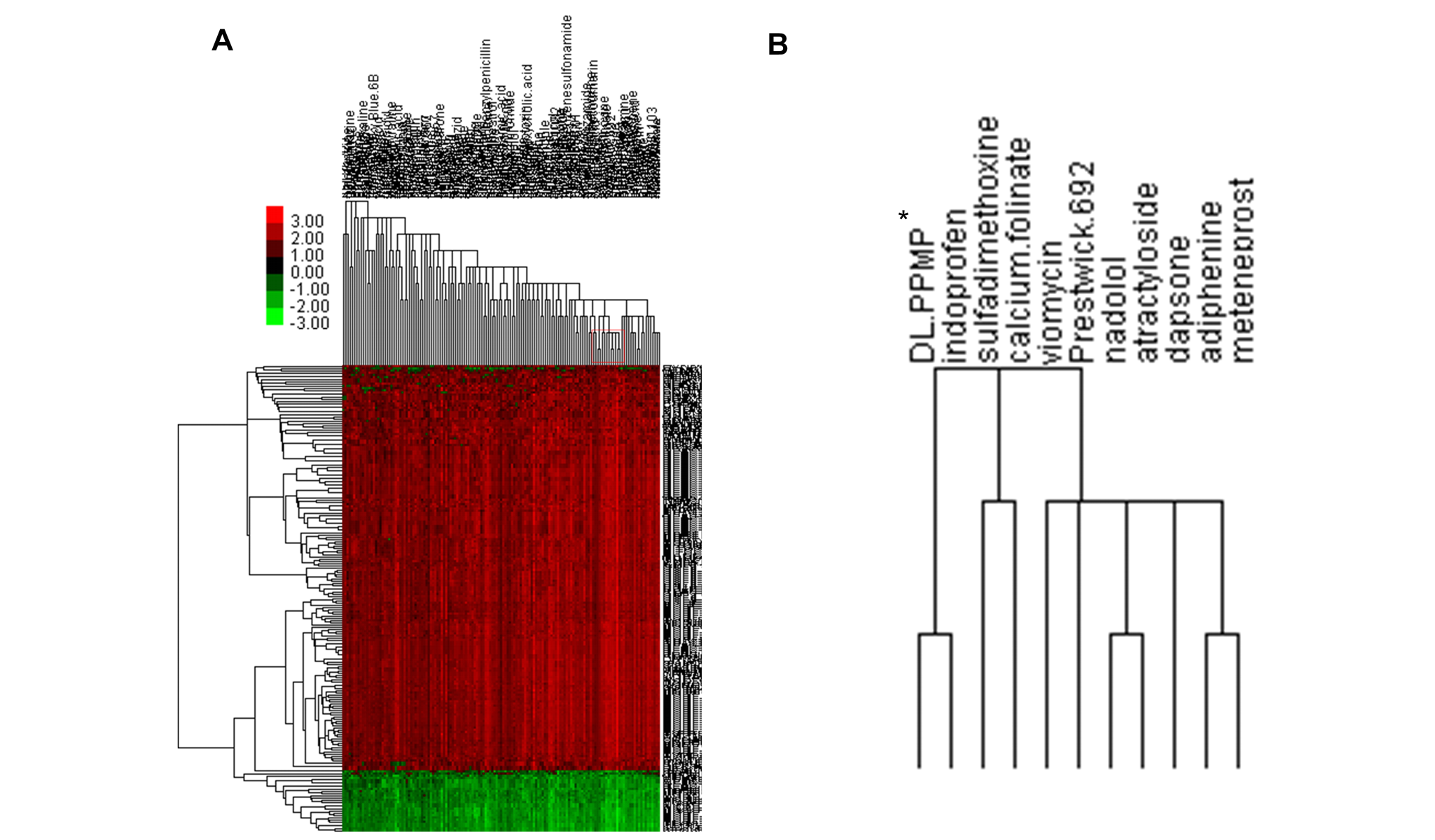

Supplement: S6 Fig — (A) A correlation > 0.9 sub-heatmap including the compound DL-PPMP of unknown function from a GSLHC-generated heatmap based on tags significantly enriched in DL-PPMP with permutation p< 0.005. (B) Detail of the dendrogram showing DL-PPMP (marked by black asterisk) with its partner drugs. (TIF) [file pone.0139889.s006.tif]

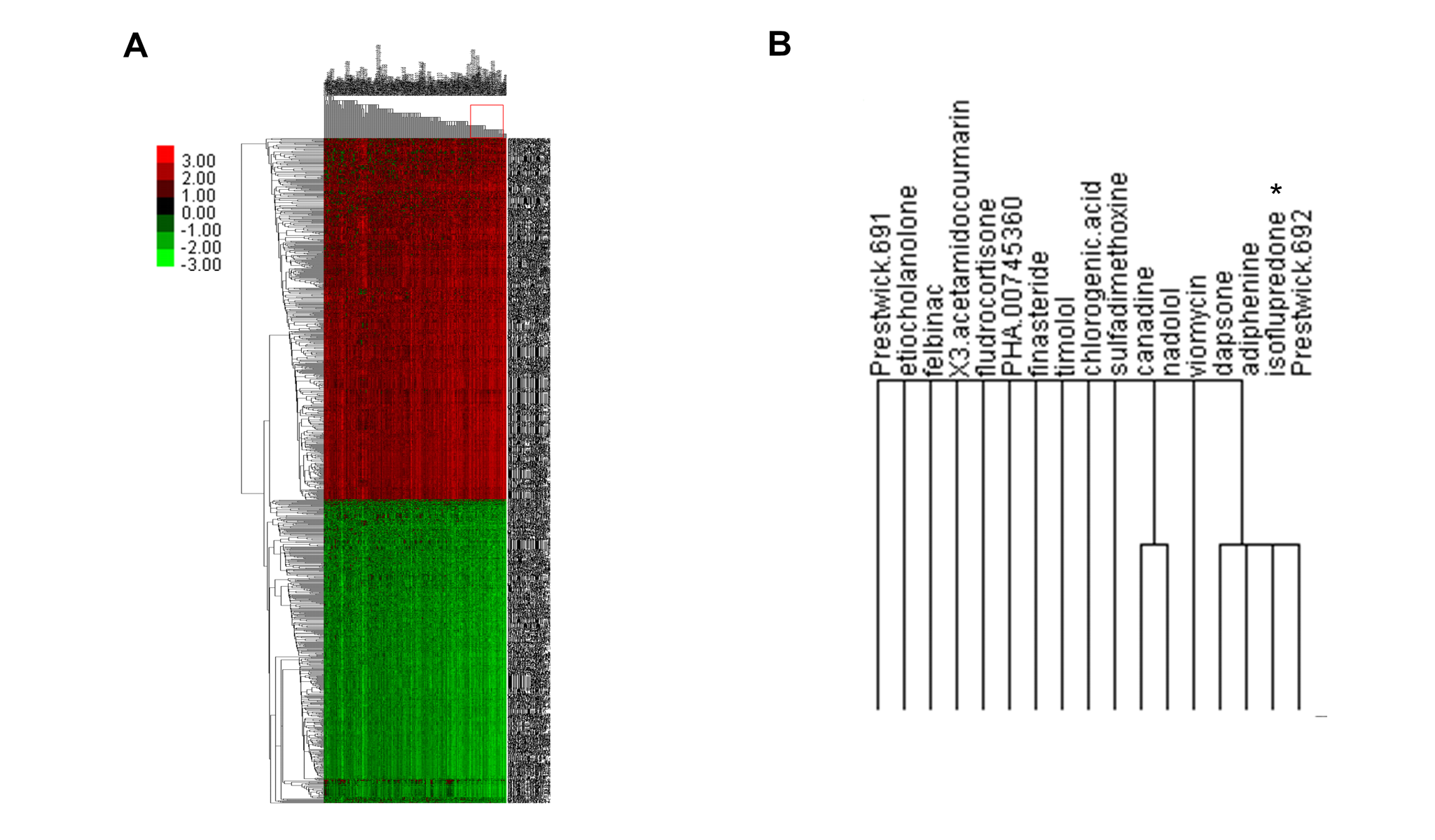

Supplement: S7 Fig — (A) A correlation > 0.9 sub-heatmap including the compound Prestwick-692 of unknown function from a GSLHC-generated heatmap based on tags significantly enriched in Prestwick-692 with permutation p< 0.005. (B) Detail of the dendrogram showing Prestwick-692 (marked by black asterisk) with its partner drugs. (TIF) [file pone.0139889.s007.tif]

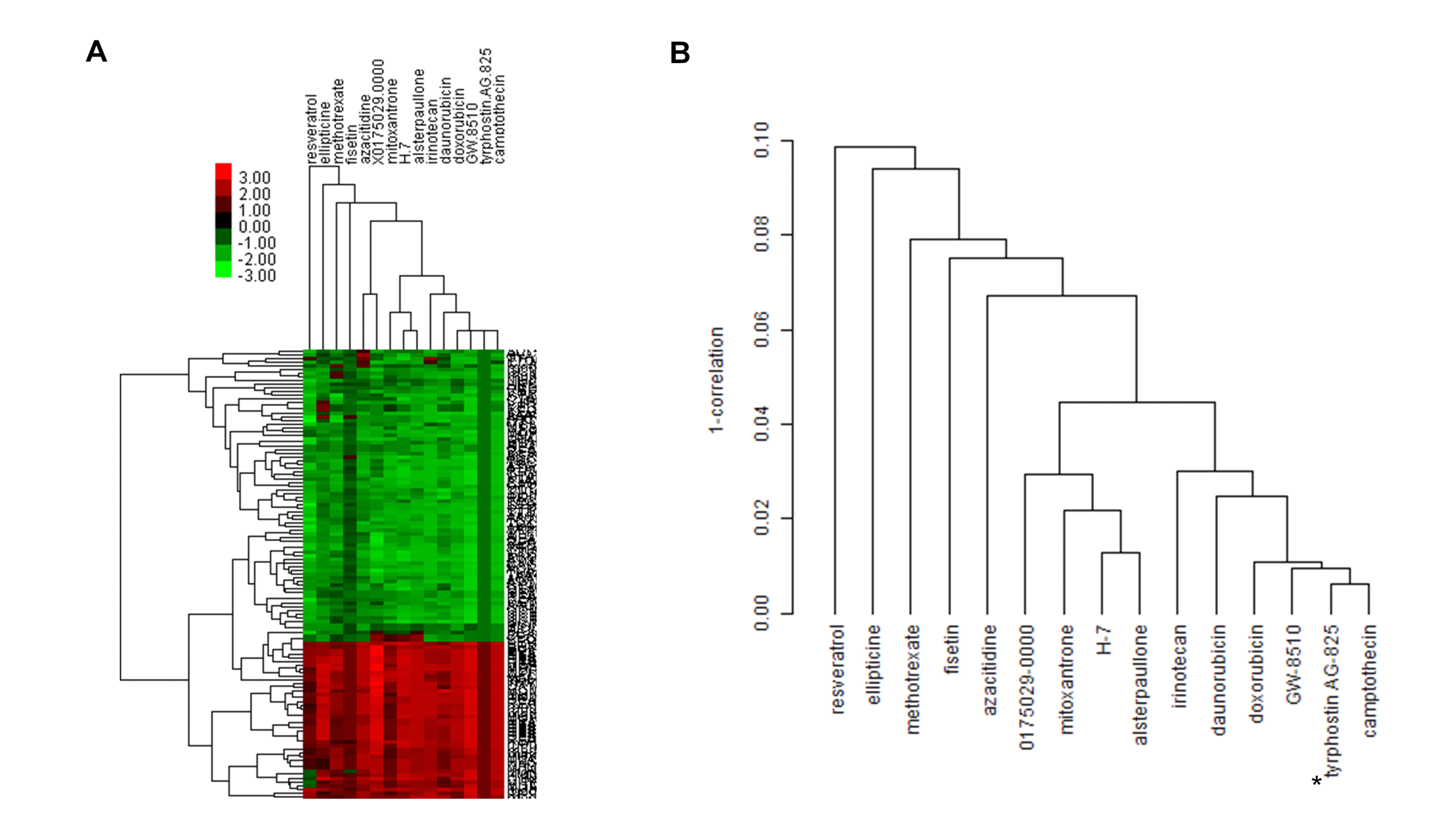

Supplement: S8 Fig — (A) A correlation > 0.9 sub-heatmap including the compound tyrphostin AG-825 of unknown function from a GSLHC-generated heatmap based on tags significantly enriched in tyrphostin AG-825 with permutation p< 0.005. (B) Detail of the dendrogram showing tyrphostin AG-825 (marked by black asterisk) with its partner drugs. (TIF) [file pone.0139889.s008.tif]

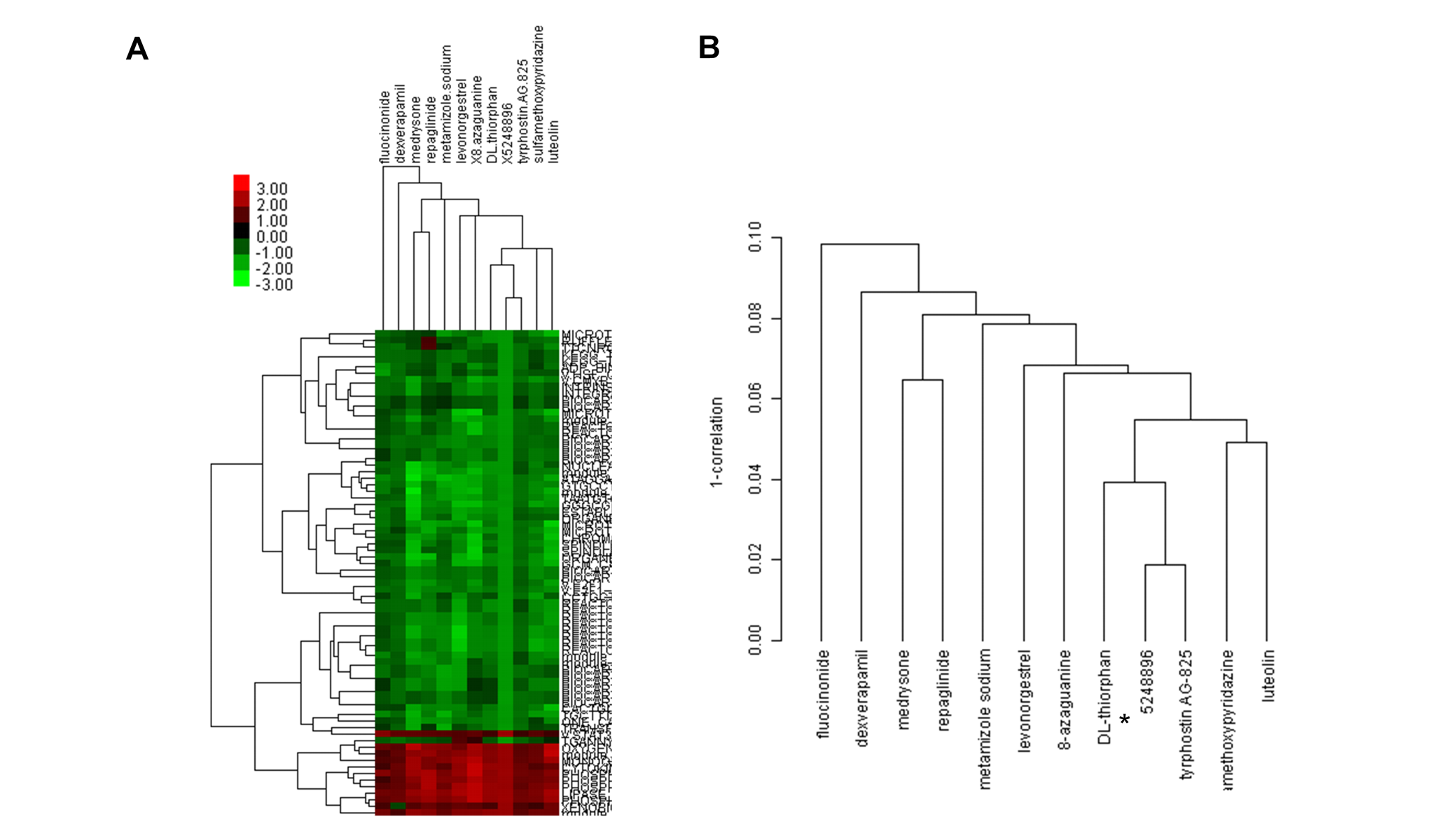

Supplement: S9 Fig — (A) A correlation > 0.9 sub-heatmap including the compound 5248896 of unknown function from a GSLHC-generated heatmap based on tags significantly enriched in 5248896 with permutation p< 0.005. (B) Detail of the dendrogram showing 5248896 (marked by black asterisk) with its partner drugs. (TIF) [file pone.0139889.s009.tif]

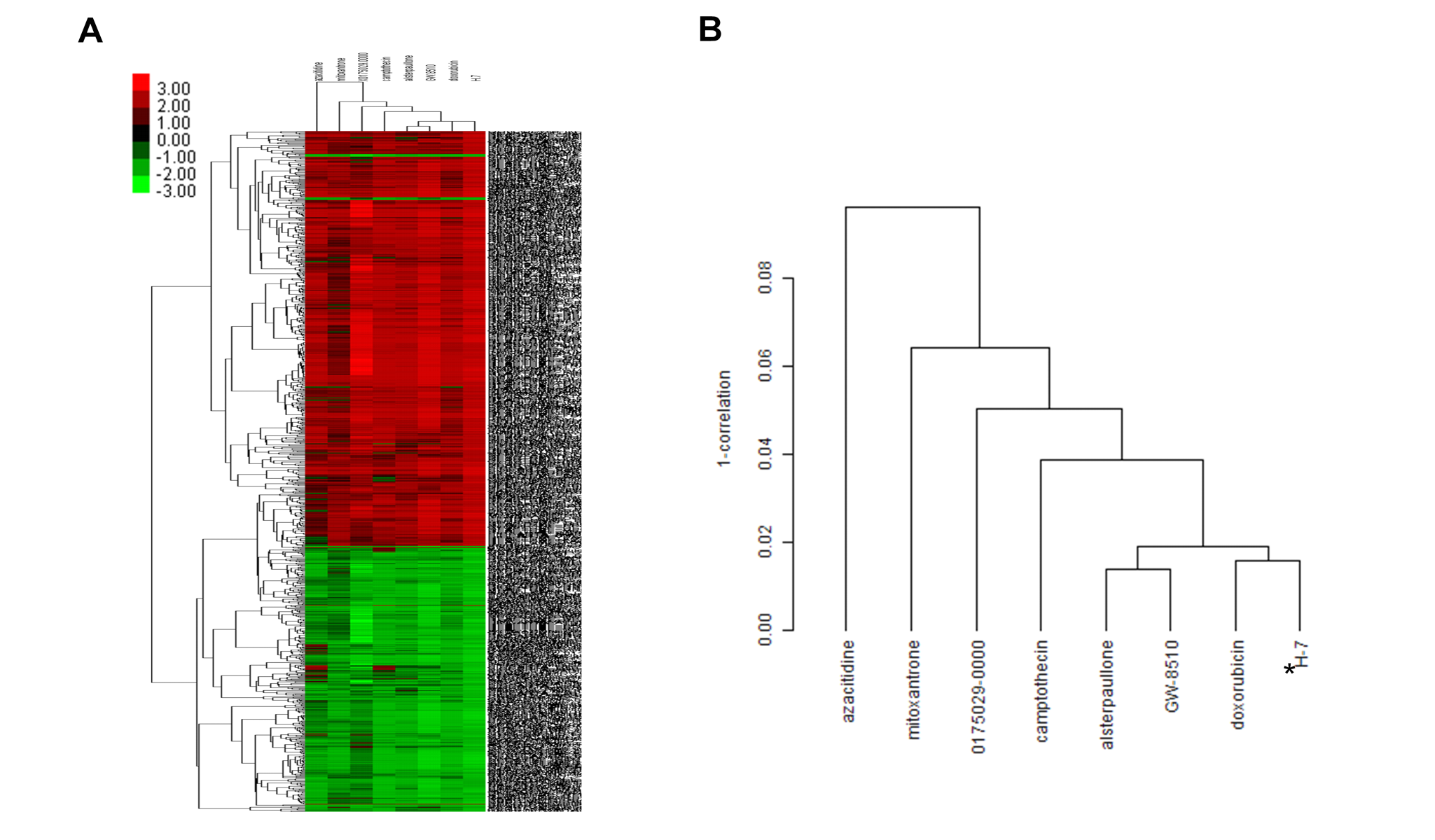

Supplement: S10 Fig — (A) A correlation > 0.9 sub-heatmap including the compound H-7 of unknown function from a GSLHC-generated heatmap based on tags significantly enriched in H-7 with permutation p< 0.005. (B) Detail of the dendrogram showing H-7 (marked by black asterisk) with its partner drugs. (TIF) [file pone.0139889.s010.tif]

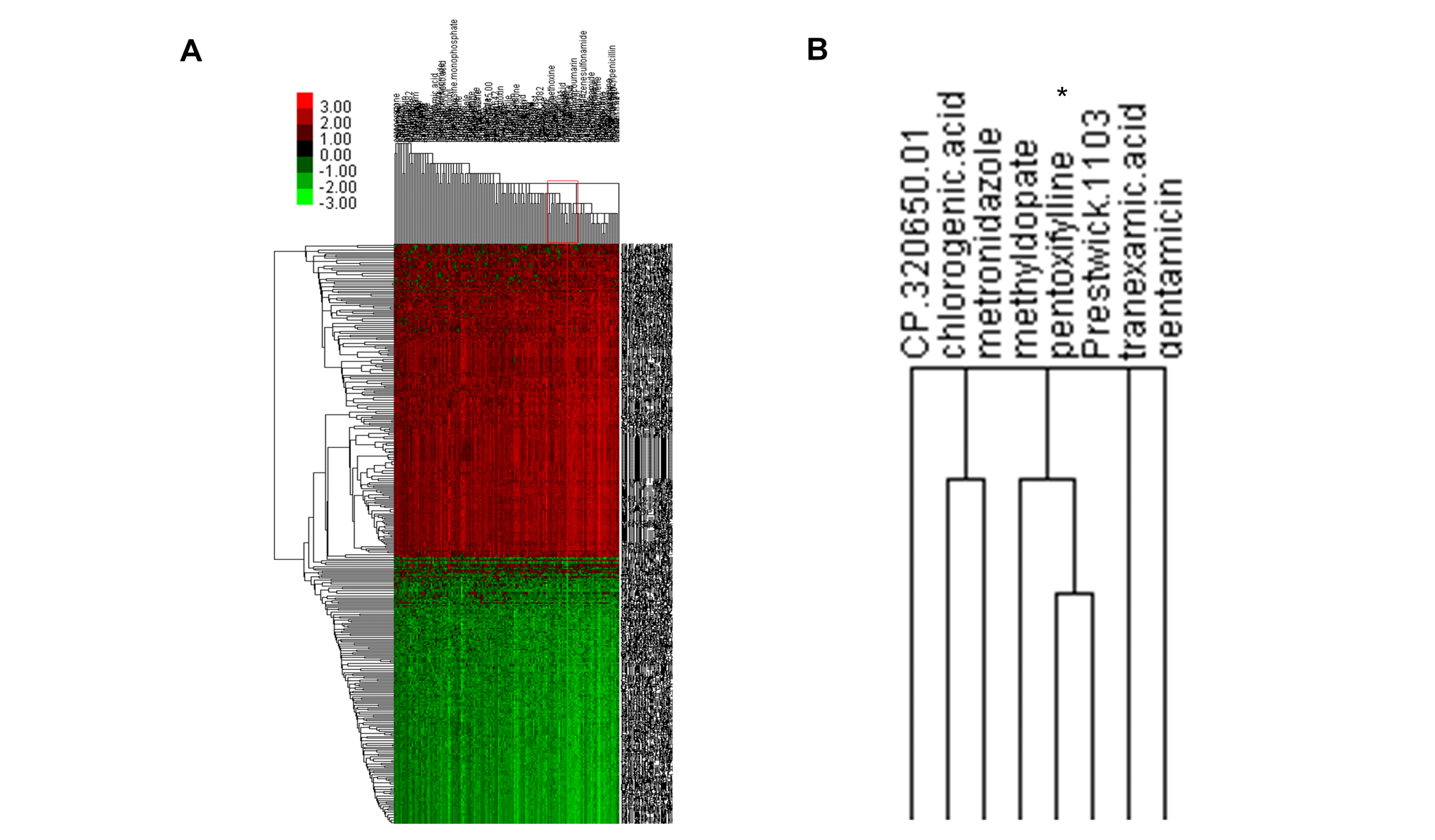

Supplement: S11 Fig — (A) A correlation > 0.9 sub-heatmap including the compound Prestwick-1103 of unknown function from a GSLHC-generated heatmap based on tags significantly enriched in Prestwick-1103 with permutation p< 0.005. (B) Detail of the dendrogram showing Prestwick-1103 (marked by black asterisk) with its partner drugs. (TIF) [file pone.0139889.s011.tif]

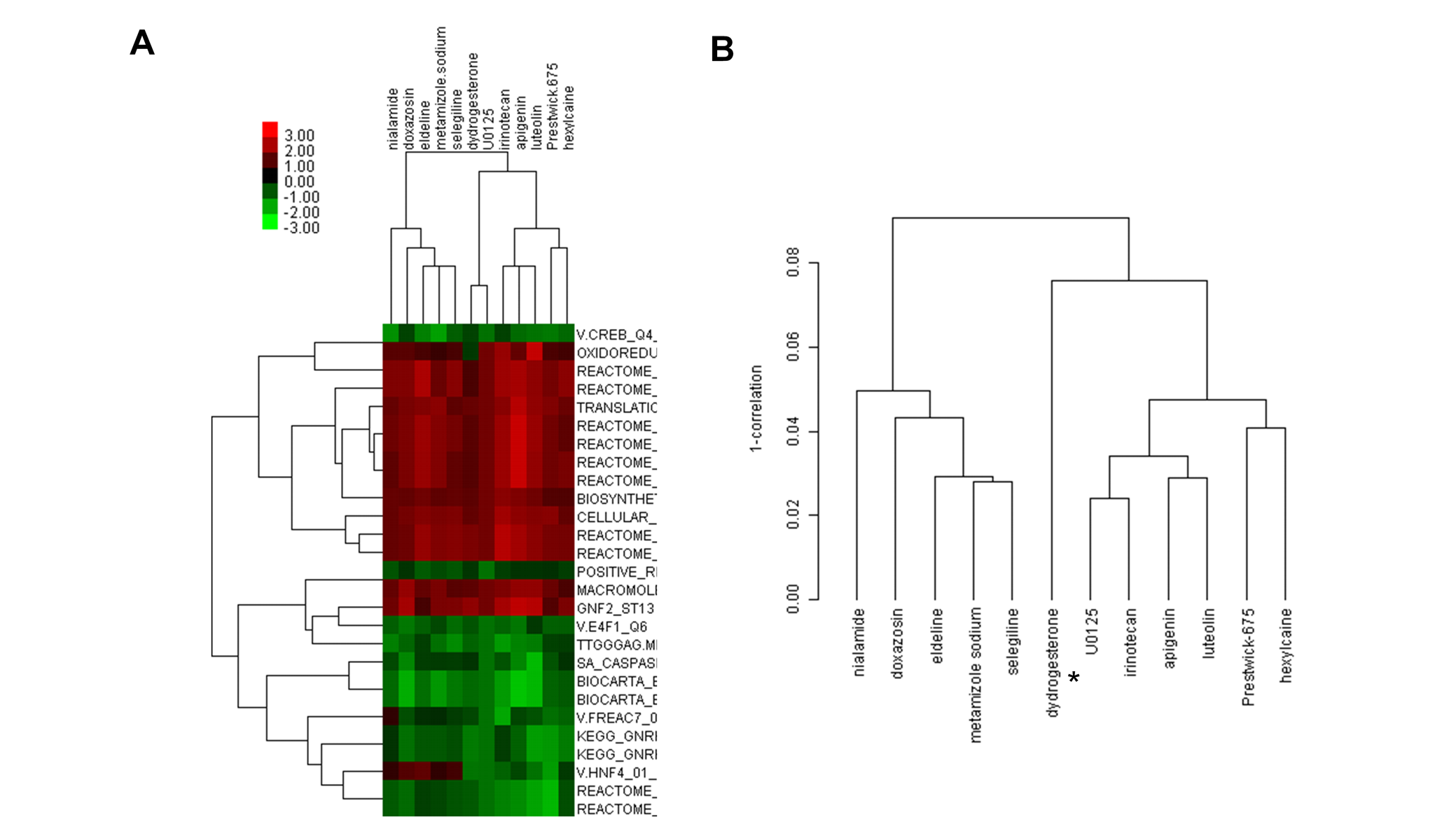

Supplement: S12 Fig — (A) A correlation > 0.9 sub-heatmap including the compound U0125 of unknown function from a GSLHC-generated heatmap based on tags significantly enriched in U0125 with permutation p< 0.005. (B) Detail of the dendrogram showing U0125 (marked by black asterisk) with its partner drugs. (TIF) [file pone.0139889.s012.tif]

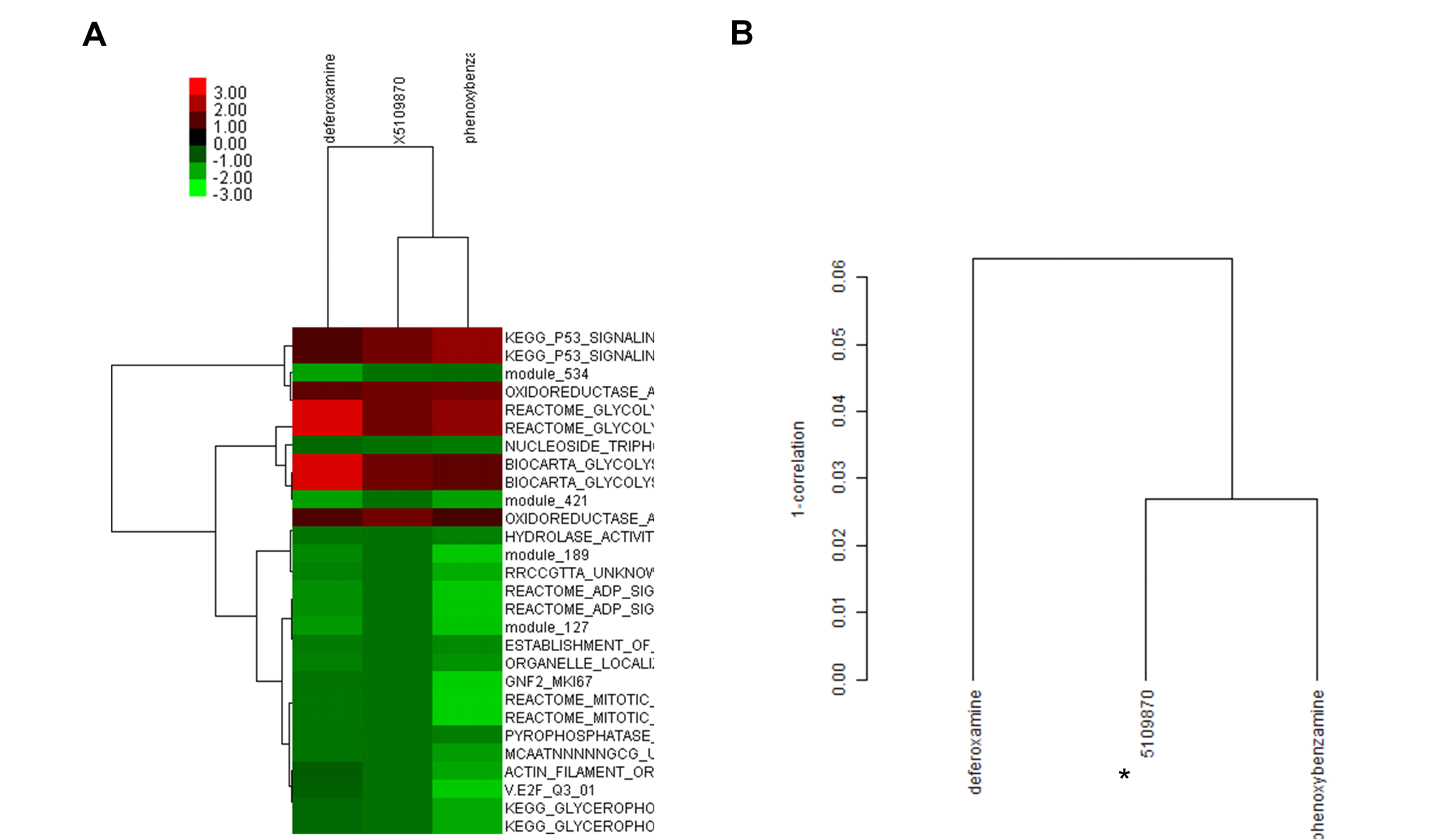

Supplement: S13 Fig — (A) A correlation > 0.9 sub-heatmap including the compound 5109870 of unknown function from a GSLHC-generated heatmap based on tags significantly enriched in 5109870 with permutation p< 0.005. (B) Detail of the dendrogram showing 5109870 (marked by black asterisk) with its partner drugs. (TIF) [file pone.0139889.s013.tif]

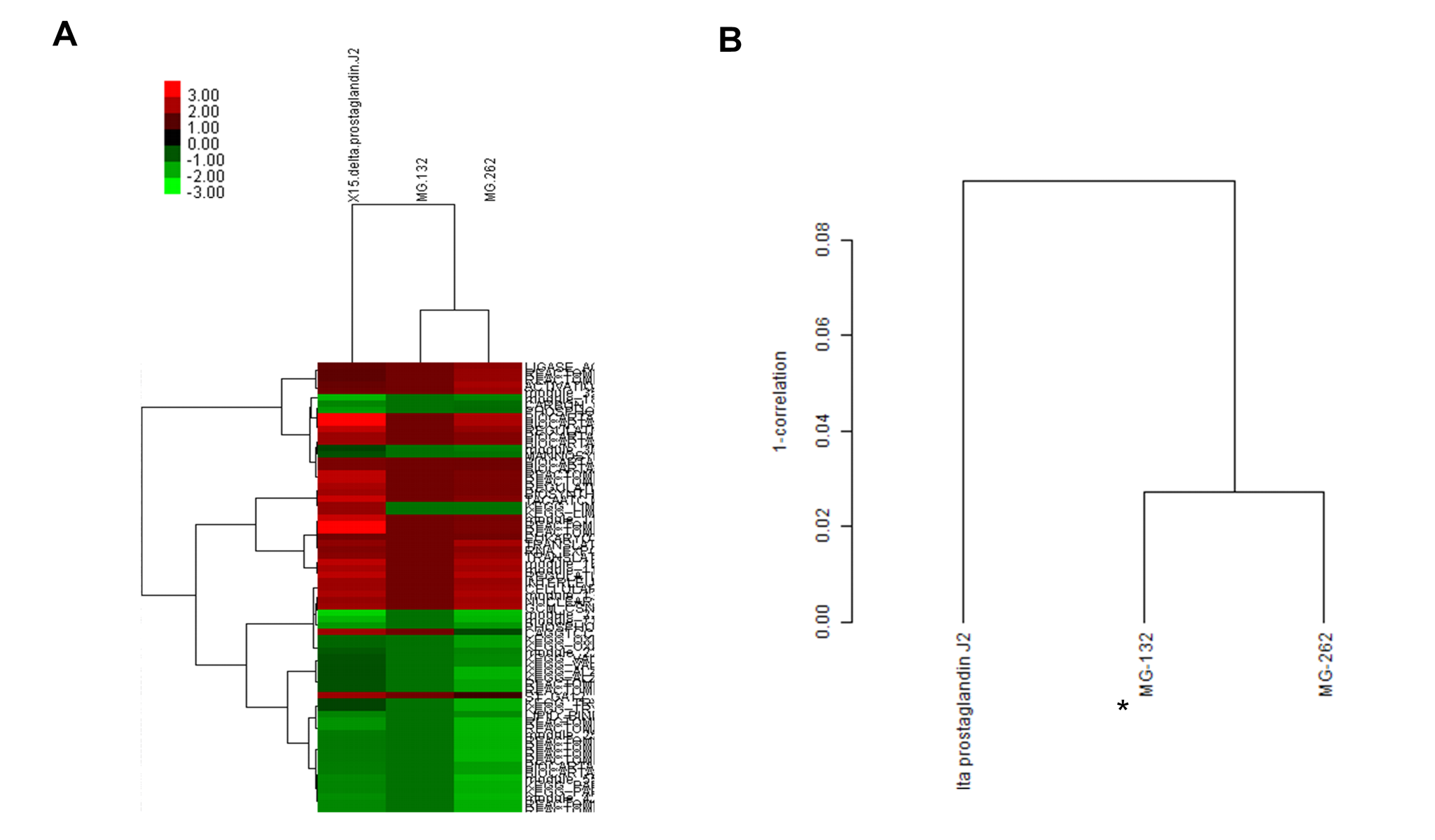

Supplement: S14 Fig — (A) A correlation > 0.9 sub-heatmap including the compound MG-132 of unknown function from a GSLHC-generated heatmap based on tags significantly enriched in MG-132 with permutation p< 0.005. (B) Detail of the dendrogram showing MG-132 (marked by black asterisk) with its partner drugs. (TIF) [file pone.0139889.s014.tif]

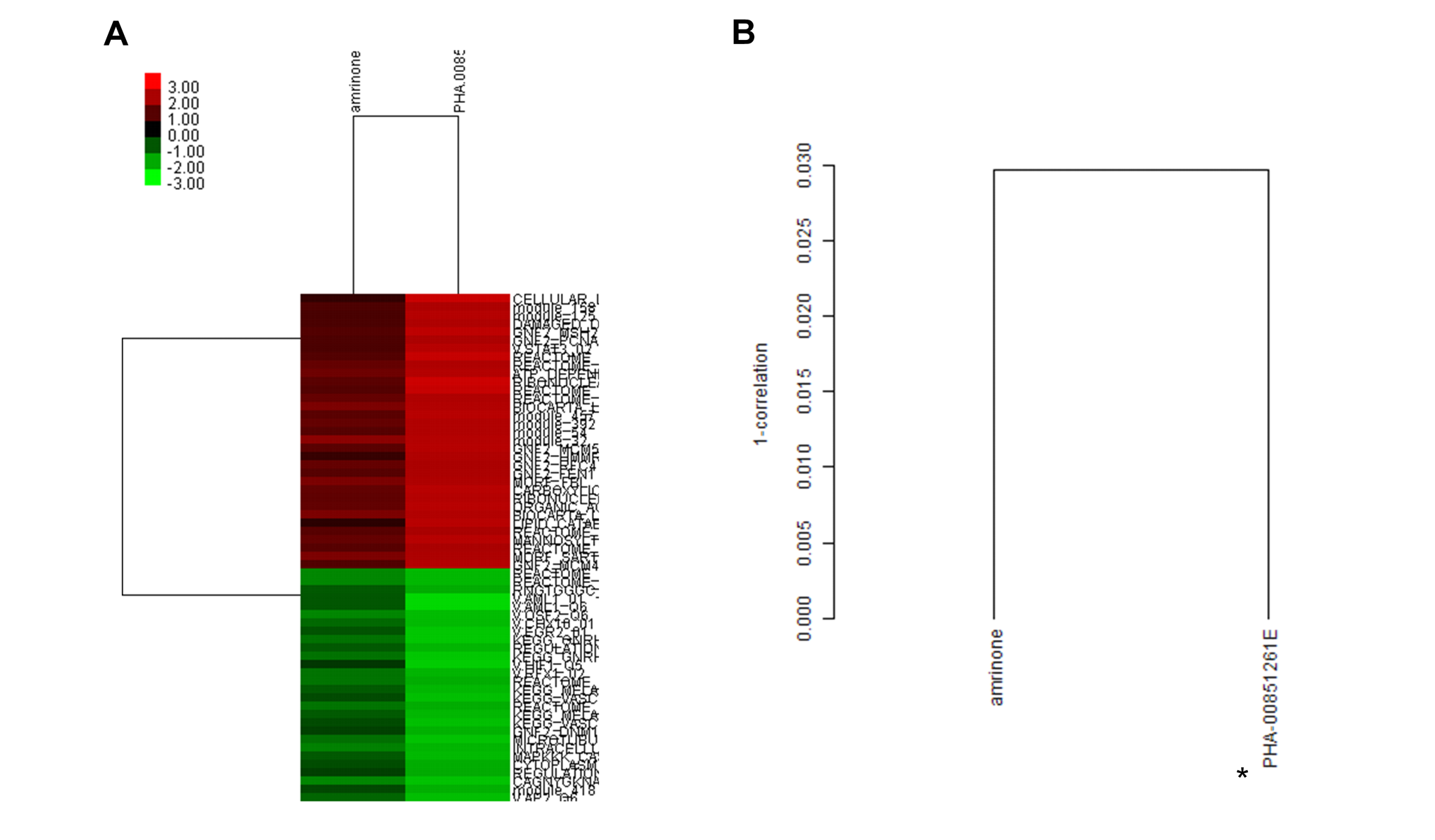

Supplement: S15 Fig — (A) A correlation > 0.9 sub-heatmap including the compound PHA-00851261E of unknown function from a GSLHC-generated heatmap based on tags significantly enriched in PHA-00851261E with permutation p< 0.005. (B) Detail of the dendrogram showing PHA-00851261E (marked by black asterisk) with its partner drugs. (TIF) [file pone.0139889.s015.tif]

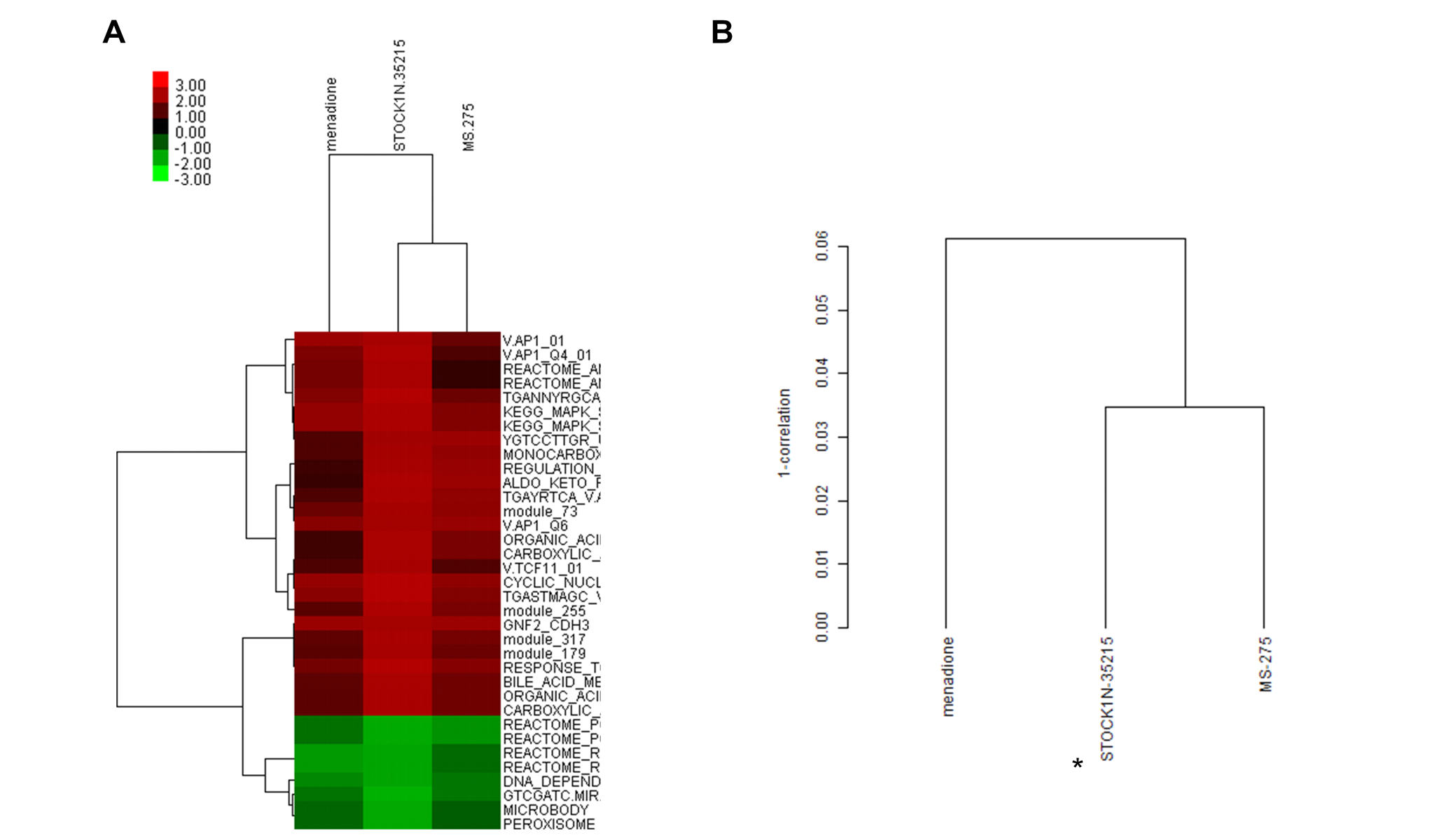

Supplement: S16 Fig — (A) A correlation > 0.9 sub-heatmap including the compound STOCK1N-35215 of unknown function from a GSLHC-generated heatmap based on tags significantly enriched in STOCK1N-35215 with permutation p< 0.005. (B) Detail of the dendrogram showing STOCK1N-35215 (marked by black asterisk) with its partner drugs. (TIF) [file pone.0139889.s016.tif]

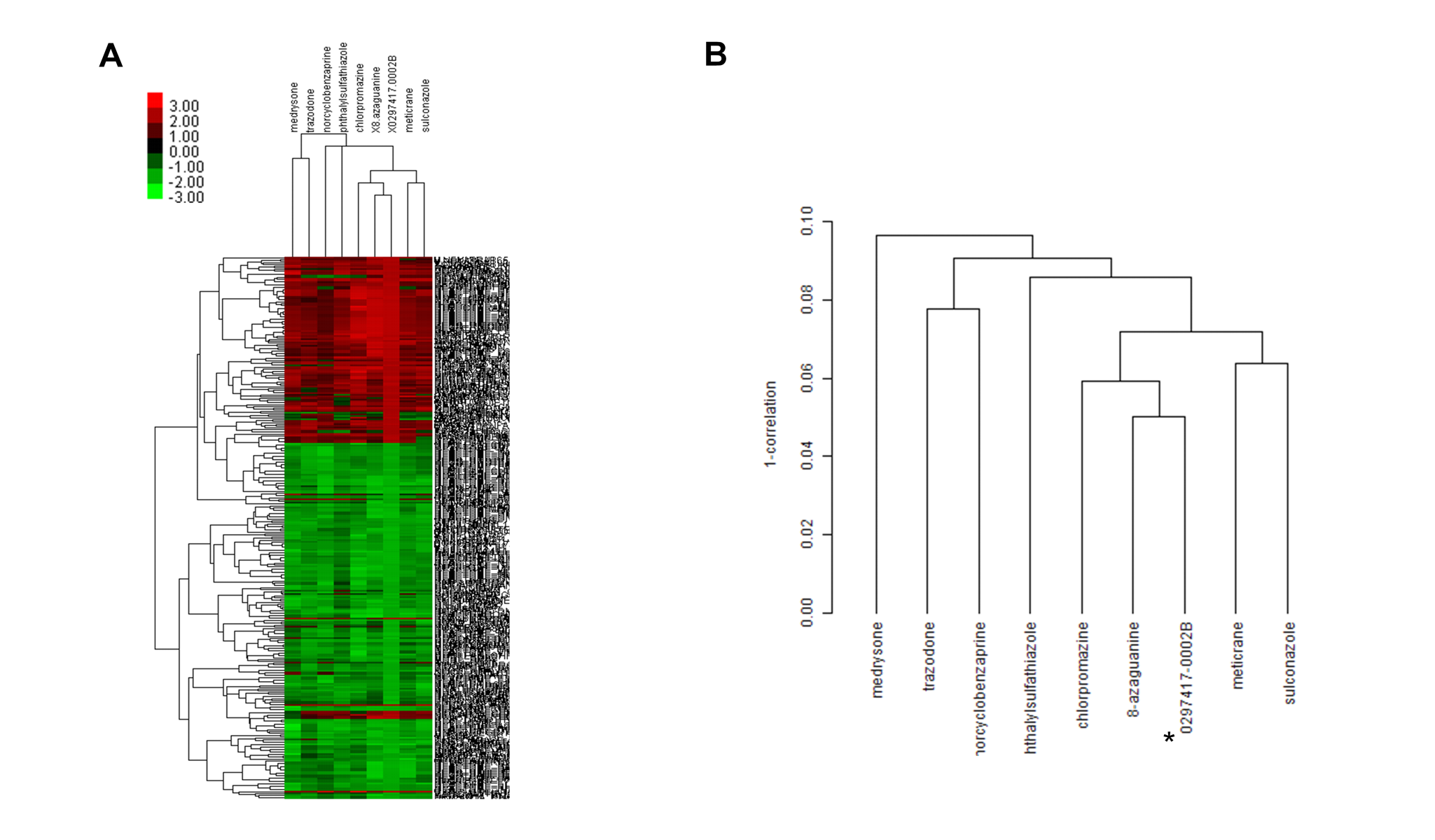

Supplement: S17 Fig — (A) A correlation > 0.9 sub-heatmap including the compound 0297417-0002B of unknown function from a GSLHC-generated heatmap based on tags significantly enriched in 0297417-0002B with permutation p< 0.005. (B) Detail of the dendrogram showing 0297417-0002B (marked by black asterisk) with its partner drugs. (TIF) [file pone.0139889.s017.tif]

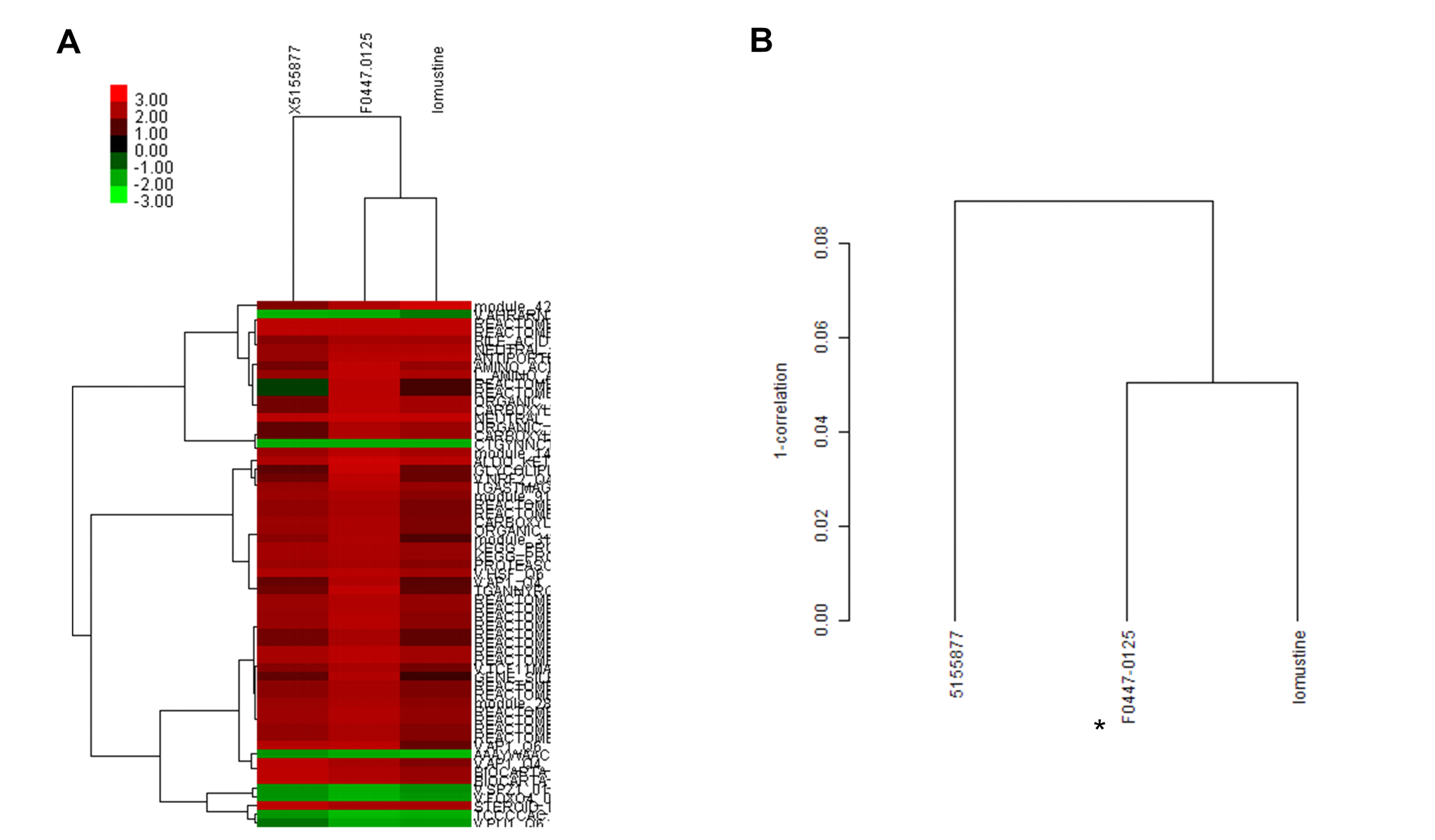

Supplement: S18 Fig — (A) A correlation > 0.9 sub-heatmap including the compound F0447-0125 of unknown function from a GSLHC-generated heatmap based on tags significantly enriched in F0447-0125 with permutation p< 0.005. (B) Detail of the dendrogram showing F0447-0125 (marked by black asterisk) with its partner drugs. (TIF) [file pone.0139889.s018.tif]

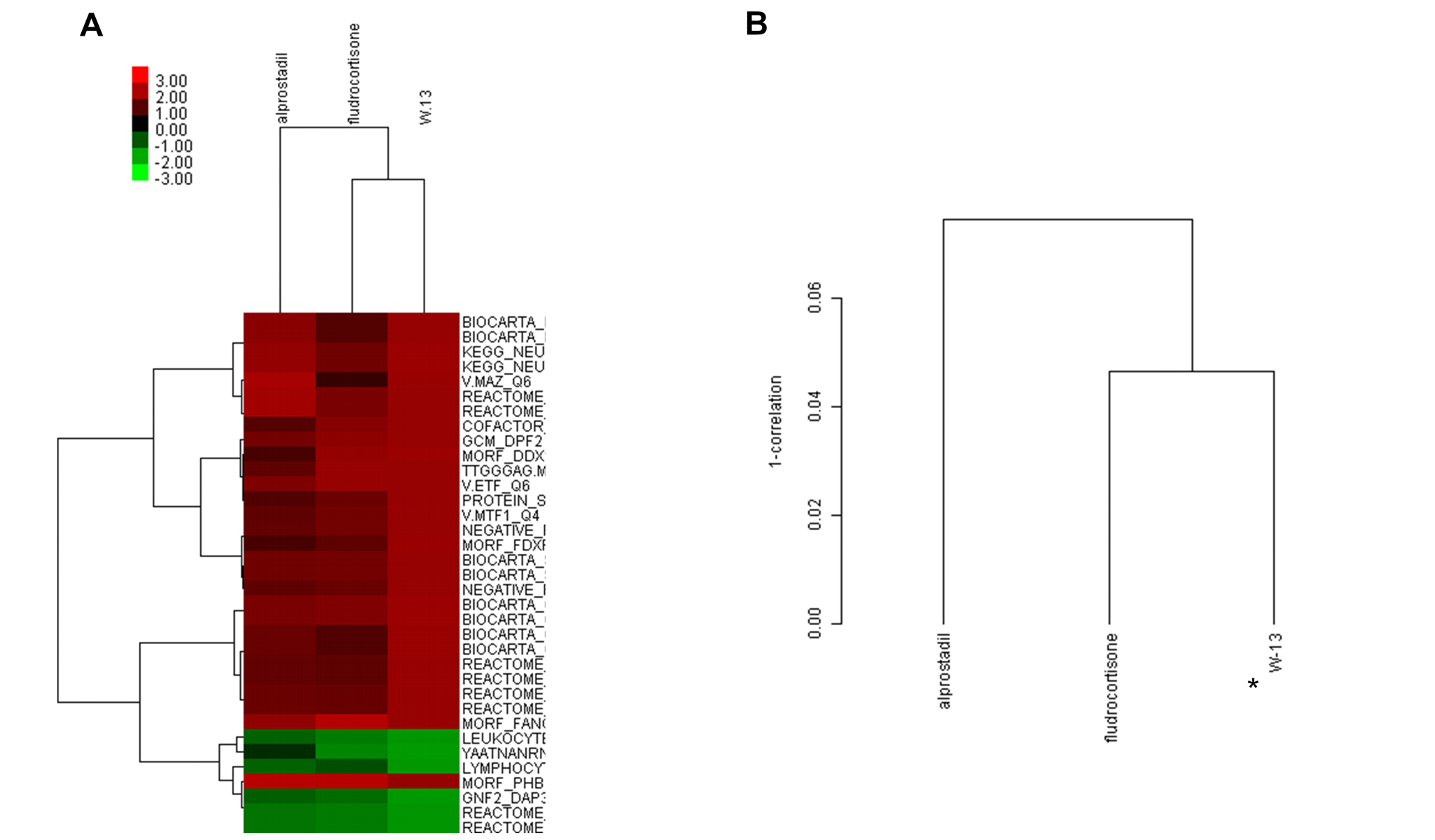

Supplement: S19 Fig — (A) A correlation > 0.9 sub-heatmap including the compound W-13 of unknown function from a GSLHC-generated heatmap based on tags significantly enriched in W-13 with permutation p< 0.005. (B) Detail of the dendrogram showing W-13 (marked by black asterisk) with its partner drugs. (TIF) [file pone.0139889.s019.tif]

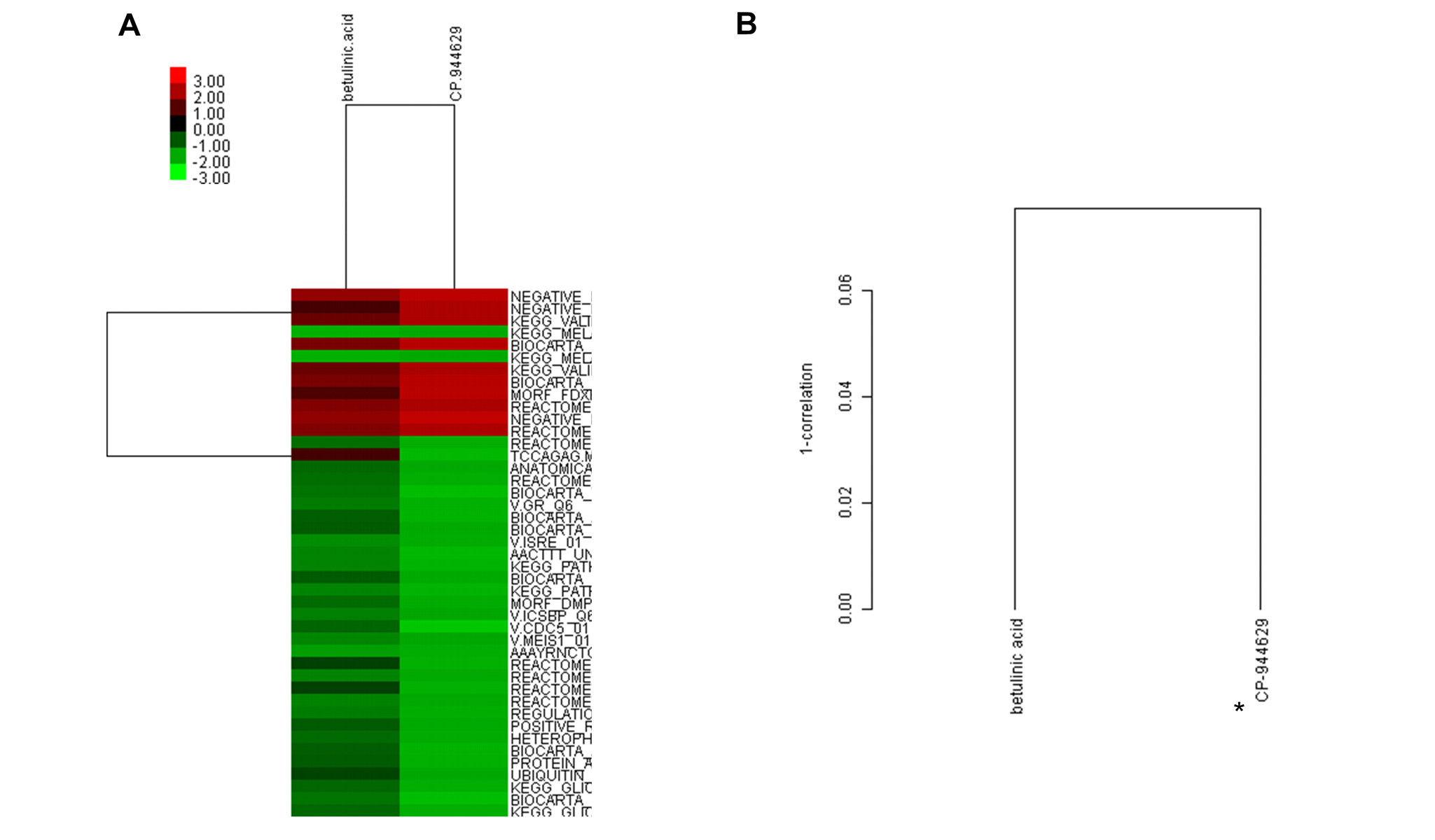

Supplement: S20 Fig — (A) A correlation > 0.9 sub-heatmap including the compound CP-944629 of unknown function from a GSLHC-generated heatmap based on tags significantly enriched in CP-944629 with permutation p< 0.005. (B) Detail of the dendrogram showing CP-944629 (marked by black asterisk) with its partner drugs. (TIF) [file pone.0139889.s020.tif]
